# Atrial myocyte senescence as a driver of atrial fibrillation: mechanisms and therapeutic implications

**DOI:** 10.3389/fcell.2025.1702207

**Published:** 2026-01-14

**Authors:** Zhaoyang Wei, Zhixiong Li, Nanhu Quan

**Affiliations:** 1 Department of Cardiology, The First Hospital of Jilin University, Changchun, Jilin, China; 2 School of Clinical Medicine, Jilin Medical University, Jilin City, Jilin, China

**Keywords:** atrial fibrillation, atrial myocytes, cell-cycle-related genes, cellular ion balance, inflammasomes, senescence

## Abstract

Atrial fibrillation (AF) is the most common type of arrhythmia encountered in the clinical setting, and its occurrence is influenced by various factors, particularly aging. Senescence of atrial myocytes plays an important role in the development of AF, although the precise mechanisms underlying this association remain unclear. This review explores the pivotal role of atrial myocyte senescence in AF pathogenesis, moving beyond chronological age. It provides evidence that aging creates a pro-arrhythmic substrate via three interconnected mechanisms: 1) inflammatory activation (mitochondrial ROS, NLRP3, and SASP), 2) dysregulated calcium handling (RyR2 and SERCA2), and 3) cell cycle disruption (p16, p21, and p53). These pathways, compounded by epigenetic changes and SIRT1/mTOR signaling dysregulation, drive the electrical and structural remodeling that triggers and sustains AF. The review highlights promising therapeutic targets such as SIRT1 activators and NLRP3 inhibitors, proposing an integrated “senescence–AF axis” model, while identifying key research gaps in cell-type specificity and clinical translation. This comprehensive review outlines current progress in research in this area and future research directions and provides valuable references for forthcoming studies.

## 1 Introduction

Atrial fibrillation (AF) is a type of cardiac arrhythmia that is commonly observed in clinical practice. It is characterized by rapid ectopic atrial impulses that replace sinus node’s control over atrial activity, leading to rapid and irregular atrial contractions. Consequently, impaired atrial contractile function manifests as palpitations, chest discomfort, and other clinical symptoms. Various factors contribute to the development of AF, including age, sex, smoking, and underlying comorbidities, with age being a particularly significant risk factor (Andrade et al., 2014; Zhang et al., 2021). It is primarily associated with cellular senescence, whereby aging cells progressively lose their regenerative capacity and accumulate cellular damage, ultimately leading to senescence of organs (van Deursen, 2014; He and Sharpless, 2017). This phenomenon also affects the heart (Shimizu and Minamino, 2019). Cardiac aging is closely linked to the aging of myocardial cells, which is defined by the irreversible loss of cellular proliferative capacity accompanied by a gradual decline in physiological function (Mao et al., 2012). Notably, most cardiomyocytes stop dividing early in life (<1% retain proliferative capacity). This suggests that cardiomyocytes may begin accumulating age-related changes from a young age. While the frailty index provides a functional measure of aging, epigenetic aging—quantified via DNA methylation—offers a molecular perspective on cellular senescence. A study utilized the frailty index, rather than chronological age, to assess cellular aging in mice. The results demonstrated positive correlations between the frailty index scores and both AF susceptibility and duration. This provides evidence that biological aging status, not just chronological age, drives AF development (Jansen et al., 2025). Thus, epigenetic age estimates cellular aging more accurately than chronological age. Unlike chronological age, which is calculated based on time, epigenetic age is determined by measuring DNA methylation levels. This provides a better representation of a cell’s biological stage. Aging of myocardial cells is influenced by various factors, including oxidative stress and the activation of intracellular inflammatory pathways (Picca et al., 2018; Zhang et al., 2022), changes in DNA methylation and cellular ion channels (Nattel et al., 2020; Kao et al., 2010), and changes in the expression levels of cell-cycle-related genes, such as p16, p53, and p21 (Kumari and Jat, 2021; Ou and Schumacher, 2018). Although these processes contribute to cardiomyocyte aging, their mechanistic links to AF initiation and progression remain poorly understood. This review proposes and elaborates on a unified conceptual framework—the senescence–AF axis—to synthesize the complex interplay between key aging mechanisms in atrial myocytes and AF pathogenesis. We posit that this axis is principally constituted by three cores, which are mutually reinforcing pathways: 1) chronic intracellular inflammatory activation (e.g., via mitochondrial ROS, NLRP3 inflammasome, and SASP), 2) dysregulation of calcium handling and ion homeostasis, and 3) disruption of cell cycle control and associated signaling (e.g., p16/p21/p53 and SIRT1/mTOR). These pathways collectively foster a pro-arrhythmic substrate characterized by electrical remodeling, structural fibrosis, and impaired cellular repair, thereby driving both the initiation and perpetuation of AF. This integrative model provides a scaffold for understanding how diverse age-related changes coalesce to promote AF and for identifying novel therapeutic targets aimed at modulating the senescence process itself, thereby offering a new perspective for geriatric cardiology and paving the way for personalized interventions targeting the biological basis of AF.

## 2 Mechanisms of initiation and maintenance of AF

AF is widely viewed to arise from ectopic depolarizations, primarily originating from the pulmonary veins but also from the vena cava and atria themselves, which override the sinoatrial node control (Brundel et al., 2022; Haissaguerre et al., 1998; Chen et al., 1999; Tsai et al., 2000). These depolarizations, which enhance local excitation of the myocardial tissue, stem from changes in the local myocardial cells exhibiting increased automaticity (Brundel et al., 2022; Ward-Caviness, 2021). While AF has traditionally been attributed to electrical remodeling, emerging evidence highlights cellular senescence as a key driver of atrial structural and functional deterioration (Chen et al., 2021b). The mechanisms of AF can be categorized as triggering or maintenance. Early and delayed after-depolarizations in the pulmonary veins may serve as the basis for this ectopic activity (Nattel et al., 2020). The precise formation of these impulses involves changes in the ion channels of atrial myocytes and the autonomic nervous system. Eliminating the excitatory connection between these vessels and the atria by ablation can effectively eliminate AF (Willems et al., 2006; Katritsis et al., 2010). While ectopic triggers initiate AF, structural remodeling and electrophysiological changes sustain it. In terms of maintenance mechanisms, some patients may not revert to sinus rhythm immediately after ablation and require electrical cardioversion, indicating the existence of mechanisms that are independent of the original ectopic impulses (Katritsis et al., 2010). Recognized maintenance mechanisms for AF include the multiple wavelet theory, reentry, and focal activity (Nattel et al., 2017).

Pathological changes, such as atrial fibrosis, impair the atrial muscle, leading to local conduction blockages. The incidence of these blockages is higher in patients with long-standing persistent AF than in those with acute AF (Allessie et al., 2010). These conduction impairments create conditions for atrial reentry, where anatomical and functional abnormalities can result in the formation of spiral waves that sustain AF (Quintanilla et al., 2021). Both the triggering and maintenance of AF involve common pathological and physiological changes, including alterations in myocardial cells, the myocardial interstitium, and the autonomic nervous system. During atrial myocyte aging, key changes such as mitochondrial dysfunction, increased reactive oxygen species (ROS), abnormal calcium channel activity, and expression of senescence-associated genes occur. These alterations closely resemble the pathological features observed in AF, suggesting a potential link between atrial myocyte aging and AF development (Chen et al., 2024; Ma et al., 2021). Together, these findings indicate that atrial myocyte senescence promotes both AF triggers (via ectopic activity) and maintenance (via structural/electrical remodeling), highlighting aging as a therapeutic target.

## 3 Senescence of atrial myocytes and its relationship with AF

While the precise triggers of cardiomyocyte senescence remain unclear, three key mechanisms have been implicated. The first involves the activation of intracellular inflammatory signaling pathways, leading to cell damage and reduced cellular protection. This includes the generation of ROS from mitochondrial dysfunction, activation of NOD-like receptor family pyrin domain-containing 3 (NLRP3) inflammasomes and the senescence-associated secretory phenotype (SASP), mammalian target of rapamycin (mTOR) signaling, and decreased sirtuin 1 (SIRT1) activity. The second mechanism involves changes in DNA methylation and cellular dysfunction resulting from cytoskeletal disintegration, primarily affecting ion channels and intracellular calcium balance. The third entails inhibition of cell proliferation-related genes, such as p53, p21, and p16. Together, these senescence mechanisms create a pro-arrhythmic substrate via structural remodeling (fibrosis), electrical dysfunction (ion channels), and impaired repair (cell cycle arrest), thus collectively promoting AF initiation and persistence. Supplementary Table S1 summarizes current research on the relationship between AF and the aging of atrial cardiomyocytes. The subsequent sections of this article integrate these findings to elucidate how the aging process influences the pathogenesis of AF.

### 3.1 Mitochondrial dysfunction and activation of intracellular inflammatory signaling

#### 3.1.1 Mitochondrial dysfunction and ROS-induced damage to atrial cells

**FIGURE 1:**
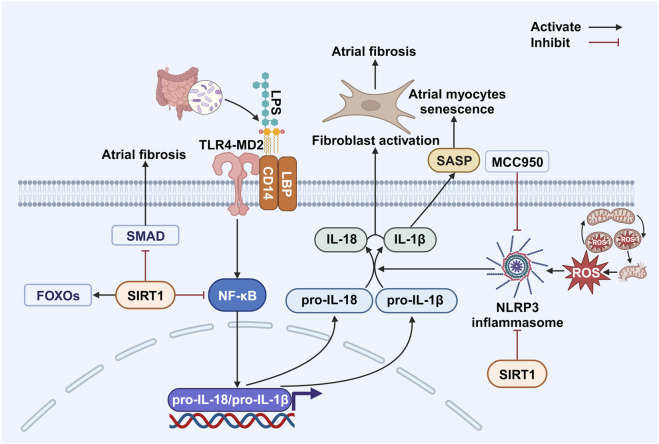
Mitochondrial dysfunction and activation of intracellular inflammatory signaling. The presence of ROS generated due to impaired mitochondrial function induces the activation of the NLRP3 inflammasome within myocardial cells. Moreover, the influence of LPS produced by the gut microbiota results in the generation of pro-IL-18 and pro-IL-1β within myocardial cells. This process ultimately culminates in cellular aging and the activation of fibroblasts. Abbreviations: CD14, cluster of differentiation 14; FOXOs, fork head box O transcription factors; IL-1β, interleukin-1β; IL-18, interleukin-18; LBP, LPS binding protein; LPS, lipopolysaccharide; NF-κB, nuclear factor-kappa B; NLRP3, NOD-like receptor family pyrin domain-containing 3; ROS, reactive oxygen species; SASP, senescence-associated secretory phenotype; SIRT1, sirtuin 1; SMAD, small mothers against decapentaplegic; TLR4-MD2, toll-like receptor 4 co-receptor myeloid differentiation protein 2. Created with BioRender.com.

Mitochondria, the primary energy source in cardiomyocytes, also generate ROS as byproducts (Kim et al., 2005). Their continuous replication throughout the cell’s lifespan can lead to mitochondrial DNA damage and mutations (Picca et al., 2018). To counteract this damage, cells activate the DNA repair enzyme poly ADP-ribose polymerase-1 (PARP-1) (Zhang et al., 2019). However, this energy-consuming repair process depletes nicotinamide adenine dinucleotide (NAD+), thereby reducing mitochondrial energy production (Zhang et al., 2019). The resulting depletion of NAD+/NADH levels impairs the electron transport chain, which increases ROS production, promotes mitochondrial DNA mutations (Picca et al., 2018; Ramos-Mondragón et al., 2023), and induces protein oxidation (Kauppila et al., 2017; van Deursen, 2014), thereby further exacerbating mitochondrial DNA damage (Zhang et al., 2019; Yu et al., 2013). These mutations, in turn, lead to increased ROS generation (Hahn and Zuryn, 2019). Furthermore, during cellular senescence, declining autophagic clearance of damaged mitochondria causes impaired energy production and excessive ROS (Chapman et al., 2019). This vicious cycle of mitochondrial dysfunction, coupled with alterations in ion channels, contributes to AF development (Zhang et al., 2019; Wiersma et al., 2019). Supporting this mechanism, studies have shown that pharmacological supplementation of NAD + or inhibition of PARP-1 can prevent impairment of atrial contractility (Ramos and Brundel, 2020). Thus, mitochondrial ROS generation creates a pro-arrhythmic environment via DNA damage, energy depletion, and ion channel dysfunction, making it a potential therapeutic target in AF (Figure 1).

#### 3.1.2 Activation of NLRP3 and AF

Building on mitochondrial ROS overproduction, aging also triggers NLRP3 inflammasome activation—a key inflammatory pathway in AF pathogenesis. Age-related gut microbiota dysbiosis increases intestinal barrier permeability (Thevaranjan et al., 2017), allowing lipopolysaccharide (LPS) from gut bacteria to enter the bloodstream and reach atrial tissue. LPS subsequently activates atrial NLRP3 inflammasomes, which promote AF occurrence (Zhang et al., 2022). This pathway is supported by evidence from animal experiments showing that elevated serum LPS contributes to AF development (Sun et al., 2016) and by clinical trials demonstrating that increased serum LPS predicts adverse events in AF patients (Pastori et al., 2017). Furthermore, comparative studies involving fecal microbiota transplantation in rodents have revealed that younger rats exhibit higher LPS levels and NLRP3 expression after transplantation than older rats. Inhibition of NLRP3 activation using MCC950 effectively suppresses atrial fibrosis and mitigates AF occurrence (Zhang et al., 2022). In summary, these findings position the LPS/NLRP3 axis as a modifiable risk pathway in age-related AF, with MCC950 demonstrating therapeutic potential (Figure 1).

LPS is transferred via LPS-binding protein and cluster of differentiation 14 to activate toll-like receptor 4 co-receptor myeloid differentiation protein 2 (TLR4-MD2) on cell membranes (Ryu et al., 2017), which subsequently activates nuclear factor-kappa B (NF-κB) within cells (Zhao et al., 2021). NF-κB translocates to the nucleus, promoting transcription of interleukin (IL)-1β/IL-18-related genes and generation of pro-IL-1β/pro-IL-18 (Ajoolabady et al., 2022). In the presence of ROS, inactive NLRP3 (which consists of leucine-rich repeat domains, a central nucleotide-binding oligomerization domain, and an amino-terminal pyrin domain) assembles with caspase-1 and the caspase recruitment domain to form the active NLRP3 inflammasome (Lamkanfi and Dixit, 2014). This complex activates caspase-1, which subsequently cleaves pro-IL-1β/pro-IL-18 into active IL-1β/IL-18 (Cordero et al., 2018; Ajoolabady et al., 2022). Extracellular IL-1β/IL-18 then activates atrial fibroblasts, thus promoting atrial fibrosis and ultimately leading to AF (Ajoolabady et al., 2022). Additionally, LPS can directly enter the cytoplasm to activate caspase-11 (Vanaja et al., 2016), leading to NLRP3-independent activation of IL-1β/IL-18, while ROS can also directly activate NLRP3 inflammasomes to promote atrial fibrosis (Kelley et al., 2019). All in all, these pathways converge on the release of IL-1β/IL-18, creating a fibrotic substrate for AF through sustained fibroblast activation (Figure 1).

#### 3.1.3 Senescence-associated secretory phenotype and aging

NLRP3-activated IL-1β promotes the production of the SASP (Figure 1), a concept first introduced by Coppe et al. (2008). SASP comprises various signaling molecules—including cytokines, chemokines, growth factors, and proteases—released by senescent cells that can impact nearby cells (Acosta et al., 2013; Coppe et al., 2010). It is associated with aging and age-related diseases (Watanabe et al., 2017; Birch and Gil, 2020) due to its link to the chronic inflammation observed in cellular senescence (Franceschi et al., 2000), a process that occurs early in patients with cardiometabolic disease and causes significant physiological damage (Liberale et al., 2020). SASP consists mainly of pro-inflammatory cytokines and extracellular matrix remodeling factors. It can reinforce cellular senescence through autocrine signaling in senescent cells themselves and induce it in neighboring cells via paracrine signaling (Gorgoulis et al., 2019; He and Sharpless, 2017; Acosta et al., 2013; Lujambio et al., 2013). This paracrine signaling may lead to abnormal senescence in previously unaffected cells, triggering additional SASP generation (Acosta et al., 2013). For example, SASP from cardiac myocytes can activate cardiac fibroblasts and impair endothelial cell function (Anderson et al., 2019). Conversely, SASP can also attract natural killer cells and macrophages, promoting the clearance of the very senescent cells that release it (Krizhanovsky et al., 2008), which is a crucial mechanism in suppressing cancer development (Eggert et al., 2016). Moreover, SASP contributes to the maintenance and intensification of inflammation, leading to chronic systemic inflammation even in the absence of disease (Ferrucci and Fabbri, 2018; Bandeen-Roche et al., 2006), which, in turn, induces senescence in other cells. Therefore, SASP may mediate the harmful effects of senescent cells in age-related diseases (Lopes-Paciencia et al., 2019; Coppe et al., 2010). Its production can be triggered by sustained DNA damage (Takahashi et al., 2012) and is also linked to the accumulation of the cGAS–STING pathway. This occurs when cytoplasmic DNA—which accumulates due to the reduced activity of cytoplasmic DNA enzymes (Takahashi et al., 2018)—activates immune responses and enhances SASP production (Gluck et al., 2017; Faget et al., 2019).

SASP is a key feature of cellular senescence that involves multiple signaling pathways. One crucial pathway is mediated by IL-1β, as evidenced by IL-1β knockout mice showing decreased SASP production (Yoshimoto et al., 2013). Senescent cells activate pathways including cGAS–STING, NF-κB, and C/EBPβ (CCAAT box/enhancer-binding protein beta), leading to the secretion of various inflammatory substances that constitute the SASP. The SASP plays a dual role, promoting both tissue healing and inflammation (Demaria et al., 2014; Coppe et al., 2008). Given its involvement in age-related diseases and tissue damage, inhibiting SASP has emerged as a therapeutic strategy. Studies have shown that SASP inhibition via the JAK/STAT pathway can reduce inflammation (Griveau et al., 2020; Wu et al., 2020) and restore insulin sensitivity (Xu et al., 2016). Similarly, inhibiting the mTOR signaling pathway with rapamycin can significantly decrease SASP levels, thereby reducing inflammation and senescence (Weichhart, 2018; Wang et al., 2017).

#### 3.1.4 Sirtuin 1 and senescence

**FIGURE 2:**
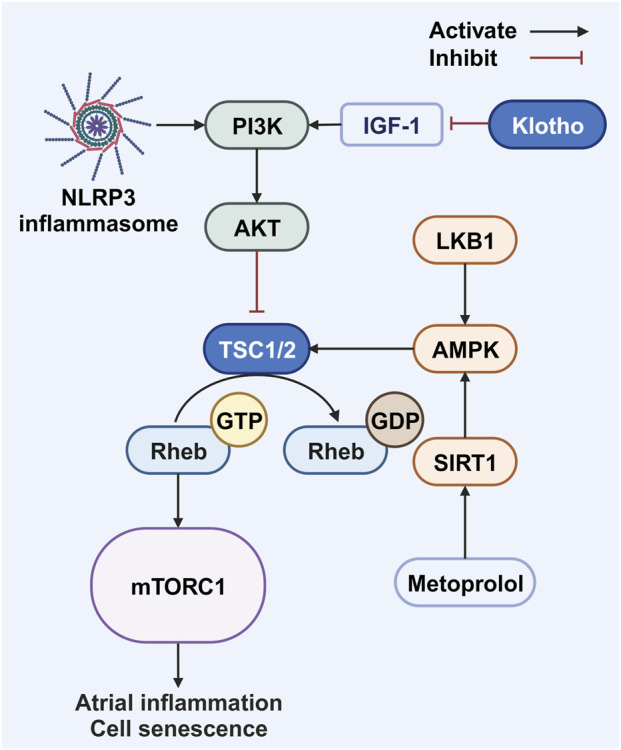
Mammalian target of rapamycin signaling pathway and senescence. Both the NLRP3 inflammasome and IGF-1 have the capacity to initiate the PI3K/AKT/mTOR pathway. However, it is noteworthy that the activity of IGF-1 is hindered by the presence of Klotho. Conversely, the activation of mTORC1 is inhibited by LKB1 and SIRT1 through the modulation of the AMPK pathway. Abbreviations: AKT, protein kinase B; AMPK, adenosine 5′-monophosphate-activated protein kinase; GDP, guanosine diphosphate; GTP, guanosine triphosphate; IGF-1, insulin-like growth factor; LKB1, liver kinase B1; mTORC1, mammalian target of rapamycin complex 1; NLRP3, NOD-like receptor family pyrin domain-containing 3; PI3K, phosphatidylinositol 3-kinase; Rheb, RAS homolog enriched in brain; SIRT1, sirtuin 1; TSC1/2, tuberous sclerosis complex 1 and 2. Created with BioRender.com.

**FIGURE 3:**
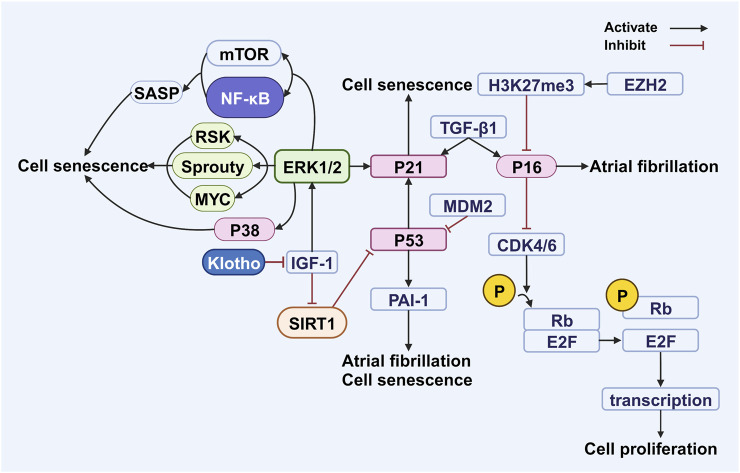
Alterations in cell-cycle-related genes and ERK signaling pathway activation. Perturbations in the expression levels of cell-cycle-related genes, namely, P16, P21, and P53, caused by various influences, including ERK1/2, TGF-β1, and SIRT1, contribute to the process of cellular senescence and serve as precursors to the onset of atrial fibrillation. Abbreviations: E2F, E2 transcription factor; ERK1/2, extracellular signal-regulated kinase 1/2; EZH2, enhancer of zeste homolog 2; H3K27me3, histone H3 lysine 27 trimethylation; IGF-1, insulin-like growth factor; mTOR, mammalian target of rapamycin; MDM2, murine double minute 2 protein; MYC, oncogenic transcription factor c-myc; PAI-1, plasminogen activator inhibitor-1; Rb, retinoblastoma protein; RSK, ribosomal S6 kinase; SASP, senescence-associated secretory phenotype; SIRT1, sirtuin 1; TGF-β1, transforming growth factor beta 1; CDK 4/6, cyclin-dependent kinase 4/6. Created with BioRender.com.

As mentioned earlier, NLRP3 activation causes inflammation in atrial cells, a process that can be inhibited by certain signaling molecules such as SIRT1. The sirtuins are a group of NAD-dependent deacetylases and ADP-ribosyltransferases (Wang et al., 2021), comprising seven known types (SIRT1–7), that primarily facilitate histone deacetylation and are linked to age-related diseases (Haigis and Sinclair, 2010; Almeida and Porter, 2019). Among them, SIRT1—an NAD + -dependent deacetylase that is highly conserved across species—has been extensively studied for its critical role in regulating aging and lifespan-associated transcription factors through substrate deacetylation (Imai et al., 2000; Chen et al., 2020; Almeida and Porter, 2019; Jin et al., 2024; Ramis et al., 2015). While different studies have reached contrasting conclusions regarding its role in promoting or reducing atrial fibrosis through different mechanisms, the evidence generally supports its protective functions. Mouse studies show that decreased SIRT1 levels can induce oxidative stress (Imai and Guarente, 2014), whereas SIRT1 activation alleviates aging-related processes, including oxidative stress, inflammation, and cell apoptosis (Campisi et al., 2019). Furthermore, SIRT1 maintains the redox balance in cardiac cells during ischemia/reperfusion, thereby delaying cellular senescence (Zhang et al., 2020), and it inhibits dynamin-related protein 1 via peroxisome proliferator-activated receptor gamma coactivator 1-alpha, thus reducing mitochondrial fission and extending cell lifespan (Ding et al., 2018). As a deacetylase, SIRT1’s active site binds NAD+ and NADH. An imbalance in the NAD+/NADH ratio prompts SIRT1 to deacetylate proteins in key signaling pathways—including adenosine 5′-monophosphate (AMP)-activated protein kinase (AMPK) (Figure 2) (Park et al., 2016), forkhead box O transcription factors (FOXOs) (Figure 1) (Jenwitheesuk et al., 2018), p53 (Figure 3) (Tran et al., 2014), NF-κB, and sirtuins (Figure 1) (Nopparat et al., 2017; Yi and Luo, 2010; Almeida and Porter, 2019)—thereby regulating cellular senescence. Through these pathways, SIRT1 exerts protective effects on myocardial cells. It is required for AMPK activation and regulates mitochondrial function via the AMPK pathway (Price et al., 2012), thereby promoting autophagy and protecting against oxidative stress (Luo et al., 2019). This mechanism is exemplified by metoprolol, which activates the SIRT1–AMPK pathway to alleviate impaired myocardial energy metabolism (Sun et al., 2017). Furthermore, SIRT1 controls FOXO transcription via histone deacetylation to prevent oxidative stress damage (Chen et al., 2021a), modulates apoptosis by inhibiting p53 and regulating p21 (Figure 3), and attenuates inflammation while reducing oxidative stress via inhibition of the NF-κB pathway (Figure 1) (Yi and Luo, 2010; Kauppinen et al., 2013; Zhou et al., 2018).

SIRT1 reduces NLRP3 inflammasome activation through the AKT signaling pathway (Han et al., 2020b). Consistently, aged NLRP3 knockout mice exhibit inhibition of the PI3K/AKT/mTOR pathway, improved autophagy, increased SIRT1 protein expression, and delayed cardiac cell senescence (Figure 2) (Marin-Aguilar et al., 2020). Furthermore, SIRT1 can alleviate cardiac fibrosis by negatively regulating the small mothers against decapentaplegic (SMAD) pathway, an anti-fibrotic effect potentially mediated through modification of SMAD2/3 and its influence on transforming growth factor beta (TGF-β) signaling (Figure 1) (Bugyei-Twum et al., 2018).

Numerous studies have established a connection between SIRT1 and AF occurrence. The nicotinamide phosphoribosyltransferase (Nampt)/NAD/SIRT1 axis, which is a key modulator of cellular senescence, is implicated in this process; decreased Nampt and NAD expressions are associated with aging and obesity, and partial Nampt deficiency in mice promotes diastolic calcium leakage and high-fat diet-induced AF (Feng et al., 2020). Furthermore, decreased SIRT1 expression has been observed in the atrial tissues of elderly humans and aged rats. Atrial-specific SIRT1 knockout mice developed atrial dilation and increased AF susceptibility. Mechanistically, acetyl-proteomics revealed that SIRT1 deficiency promotes receptor-interacting protein kinase 1 acetylation, triggering mixed-lineage kinase domain-like protein phosphorylation and atrial necroptosis. The necroptosis inhibitor necrosulfonamide reversed atrial remodeling and AF susceptibility in SIRT1-deficient mice, while resveratrol prevented age-related AF by activating SIRT1 and suppressing necroptosis (Jin et al., 2024). Elevated plasma homocysteine levels also contribute to atrial fibrosis via interaction between transient receptor potential cation channel subfamily C3 (TRPC3) and SIRT1, further underscoring that SIRT1 downregulation is a contributing factor in AF (Han et al., 2020a). Various agents, including fenofibrate, Dingjifumai Tang, resveratrol, curcumin, naringenin, and SRT1720, have been identified as SIRT1 activators (Liu et al., 2016; Liang et al., 2021; Howitz et al., 2003; Xiao et al., 2016; Testai et al., 2020; Mitchell et al., 2014).

However, research on whether SIRT1 activation reduces AF occurrence has yielded conflicting results. A study investigating the effect of B-type natriuretic peptide (BNP) on atrial fibrosis in mice found that tumor necrosis factor-alpha enhances fibrosis by increasing matrix metalloproteinase-2 (MMP-2) expression and collagen accumulation (Tsai et al., 2019). Since BNP also stimulates MMP-2 expression in human atrial myofibroblasts and SIRT1 inhibition significantly reduces this BNP-induced expression, these findings suggest SIRT1 may mediate BNP’s regulation of MMP-2 (Tsai et al., 2019). This implies that SIRT1 activation could potentially promote atrial fibrosis, highlighting the need to clarify its association with AF.

#### 3.1.5 mTOR signaling pathway and senescence

In addition to activating caspase-1, NLRP3 activates the mTOR signaling pathway. Rapamycin, an antifungal antibiotic extracted from *Streptomyces hygroscopicus* (Vezina et al., 1975), was later found to selectively inhibit the mTOR protein—a serine/threonine kinase of the PI3K-related kinase family (Keith and Schreiber, 1995)—leading to its designation as the ‘mammalian target of rapamycin’ (Brown et al., 1994; Sabatini et al., 1994). mTOR exists in two complexes, mTORC1 and mTORC2. Research across various organisms, including nematodes, fungi, and insects, has associated mTOR activation with aging, while its inhibition extends lifespan (Vellai et al., 2003). This is also observed in mammals, where depleting or inhibiting mTORC1 prolongs the lifespan and maintains health (Wu et al., 2013; Bitto et al., 2016). Mechanistically, mTOR inhibition restores age-related declines in autophagy (Hansen et al., 2018), and mTORC1 promotes stem-cell exhaustion (Yilmaz et al., 2012) and contributes to the functional decline of healthy tissues (Nacarelli et al., 2016; Herranz et al., 2015; Laberge et al., 2015). Although rapamycin has been used to extend the lifespan in model organisms (Bjedov et al., 2010; Robida-Stubbs et al., 2012), its clinical anti-aging application is limited by adverse effects. Intermittent administration may help mitigate these (Arriola Apelo et al., 2016a; Arriola Apelo et al., 2016b), and low-dose rapamycin may even exert immunoenhancing effects despite its immunosuppressant properties (Figure 2) (Mannick et al., 2018).

mTOR is linked not only to aging but also to AF occurrence. Activation of the AKT/mTOR pathway was observed in a rodent AF model (Zou et al., 2016), and gene set enrichment analysis of human atrial tissue confirmed the mTOR pathway’s association with AF development, although a causal relationship requires further validation (Ebana et al., 2019). mTOR is believed to promote AF through inflammation and structural remodeling. In human AF models, atrial cells show increased signaling in pathways such as mitogen-activated protein kinase and mTOR (Wei et al., 2020). In mice, NLRP3 inhibition suppresses the PI3K/AKT/mTOR pathway, thus protecting cardiac function and extending lifespan (Marin-Aguilar et al., 2020). Similarly, while insulin-like growth factor 1 (IGF-1) activates the PI3K pathway, Klotho inhibits it, thereby reducing mTOR activation (Dalton et al., 2017; Kurosu et al., 2005). Furthermore, inhibiting the ERK1/2 (extracellular signal-regulated kinase 1/2) and AKT/mTOR pathways in AF mice reduces the recruitment of CD3^+^ T lymphocytes and F4/80+ macrophages, thereby mitigating atrial inflammation and structural remodeling (Liu et al., 2021). Loss of liver kinase B1 (LKB1) in mice leads to heart failure and AF, accompanied by increased mTOR phosphorylation and alterations in the AMPK and mTOR/p70S6K/eEF2 pathways that contribute to cardiac hypertrophy and dysfunction (Figure 2) (Ikeda et al., 2009).

### 3.2 DNA methylation and changes in intracellular ion balance

#### 3.2.1 DNA methylation

DNA methylation is a vital epigenetic mechanism for gene regulation and serves as an important determinant of an organism’s epigenetic age (Lee et al., 2016). It involves the addition of methyl groups to specific nucleotides, typically cytosine in cytosine–phosphate–guanine islands, without altering the DNA sequence (Movassagh et al., 2011; Zhang et al., 2018). This process is catalyzed by DNA methyltransferases, which transfer methyl groups from S-adenosylmethionine to the 5′carbon of cytosine (Hannum et al., 2013; Simpkin et al., 2016). DNA methylation alters gene expression by modifying DNA-binding proteins and chromatin structure (Unnikrishnan et al., 2019), primarily silencing gene promoters and regulatory regions (Donate Puertas et al., 2017). Its effect depends on the location: high promoter methylation inhibits transcription, while low promoter methylation promotes it (de Mendoza et al., 2022). During aging, global cytosine–phosphate–guanine methylation decreases outside promoter regions, while methylation levels at promoter regions increase (Day et al., 2013; Calvanese et al., 2009).

**FIGURE 4:**
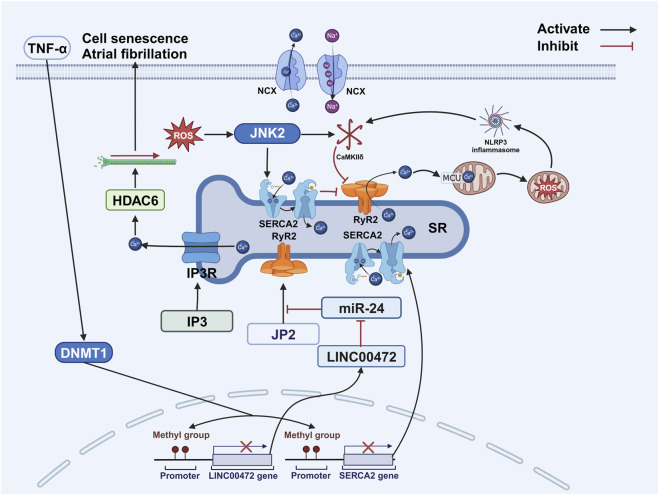
DNA methylation and changes in intracellular ion balance. DNA methylation interferes with the expression of genes associated with LINC00472 and SERCA2, leading to diminished quantities of sarcoplasmic reticulum-resident RYR2 and SERCA2. Consequently, this disruption gives rise to an altered equilibrium of cellular calcium ions, thereby rendering myocardial cells more susceptible to excitatory stimuli, ultimately promoting the occurrence of atrial fibrillation. Abbreviations: CaMKIIδ, calcium/calmodulin-dependent protein kinase II delta; DNMT1, DNA methyltransferase 1; ERK1/2, extracellular signal-regulated kinase 1/2; HDAC6, histone deacetylase 6; IP3, inositol 1,4,5-trisphosphate; JNK2, JUN N-terminal kinase 2; JP2, junctophilin-2; MCU, mitochondrial calcium uniporter; NCX, sodium–calcium exchanger; miR-24, miRNA-24; NLRP3, NOD-like receptor family pyrin domain-containing 3; ROS, reactive oxygen species; RyR2, ryanodine receptor 2; SERCA2, sarcoplasmic/endoplasmic reticulum calcium ATPase 2; SR, sarcoplasmic reticulum; TNF-α, tumor necrosis factor-alpha. Created with BioRender.com.

DNA methylation regulates the expression of calcium handling proteins and contributes to the development of AF. For instance, tumor necrosis factor-alpha reduces the expression of sarcoplasmic/endoplasmic reticulum calcium ATPase 2 (SERCA2)—a protein responsible for transporting calcium ions into the sarcoplasmic reticulum during cardiac myocyte relaxation—via DNA methyltransferase-1-mediated promoter methylation (Kao et al., 2010). Studies suggest that JUN N-terminal kinase 2 activates SERCA2, thereby enhancing sarcoplasmic reticulum calcium uptake (Yan et al., 2021). Furthermore, DNA methylation affects ryanodine receptor 2 (RyR2) expression. In AF patients, increased methylation in the promoter of the long non-coding RNA LINC00472 elevates the levels of competing RNA miR-24, which directly binds and negatively regulates both LINC00472 and junctophilin-2. Reduced junctophilin-2 expression disrupts RyR2 function and is associated with AF occurrence (Figure 4) (Wang et al., 2019).

#### 3.2.2 Alterations in calcium-handling proteins and the occurrence of ectopic excitation

The mechanism triggering AF involves abnormal stimulation of atrial myocardial cells and extends to connected blood vessels, a process associated with changes in ion channels (Nattel et al., 2007; Christ et al., 2004). Key ion channels impacting atrial impulse generation include sodium, potassium, and calcium channels, with age-related changes in calcium channels playing a crucial role in ectopic activity (Dridi et al., 2020; Christ et al., 2004). Myocardial contraction relies on calcium-induced calcium release from the sarcoplasmic reticulum (Figure 4). During contraction, calcium influx through L-type channels activates RyR2 channels on the sarcoplasmic reticulum, triggering a rapid calcium release that drives contraction (Bers, 2014). During diastole, RyR2 channels are normally closed, and intracellular calcium is sequestered by SERCA2 or extruded via the sodium–calcium exchanger (Bers, 2014). The molecular mechanism for RyR2 generating abnormal impulses involves diastolic calcium leakage from the sarcoplasmic reticulum (Nattel et al., 2020). In AF-susceptible individuals, ROS-induced sarcoplasmic reticulum stress can activate JUN N-terminal kinase (Davis, 2000), which, in turn, activates calcium/calmodulin-dependent protein kinase II delta (CaMKIIδ), promoting RyR2 dysfunction (Chiang et al., 2014; Yan et al., 2018b) and diastolic calcium leakage (Yan et al., 2021; Yan et al., 2018b). A human study corroborated this, finding that senescent CD8^+^ T cells in AF patients’ left atrial appendage secrete interferon-γ, activating the CaMKII/RyR2 pathway. This increased sarcoplasmic reticulum calcium leakage caused action potential alternans, significantly decreased conduction velocity, and markedly prolonged AF induction and duration, suggesting that RyR2-mediated leakage contributes to AF development (Xiang et al., 2024). RyR2 channel leakage during cardiac diastole elevates cytosolic calcium concentration in myocardial cells, thereby increasing myocardial excitability and contributing to arrhythmias (Bers, 2014; Yan et al., 2021). Atrial myocardial cells exhibit stronger SERCA and NCX functionality than ventricular cells, leading to higher sarcoplasmic reticulum calcium levels (Walden et al., 2009). However, this finding stems from rodent studies, and given the species’ differences in calcium handling dependence, it is uncertain whether it fully translates to human cardiomyocytes. When stimulated, RyRs become more prone to spontaneous calcium leakage (Chelu et al., 2009; Venetucci et al., 2008). This leaked calcium can bind the mitochondrial calcium uniporter, leading to mitochondrial calcium accumulation, ROS release, and subsequent NLRP3 inflammasome activation (Masters et al., 2010). The activated NLRP3 inflammasome then enhances CaMKIIδ-dependent RyR2 phosphorylation and pro-arrhythmic calcium activity (Heijman et al., 2020; Chelu et al., 2009; Neef et al., 2010). Animal experiments confirm that this CaMKIIδ-mediated calcium dysregulation underlies AF (Li et al., 2014). Although these calcium channel alterations collectively contribute to AF, caution is warranted when extrapolating these findings to humans pending clinical confirmation (Figure 4).

Phosphorylation of striated preferentially expressed protein kinase reduces RyR2-mediated release of calcium from the sarcoplasmic reticulum, and decreased phosphorylation of striated preferentially expressed protein kinase promotes the occurrence of AF (Campbell et al., 2020). Therefore, RyR2 not only influences the initiation of AF but also its maintenance.

#### 3.2.3 Changes in intracellular cytoskeleton structure

As mentioned earlier, sarcoplasmic reticulum stress increases calcium accumulation in cardiac myocytes. This elevated intracellular calcium concentration induces alterations to the cytoskeleton in atrial myocytes. The cytoskeleton, composed of microtubules, microfilaments, and intermediate filaments, provides structural support, determines organelle distribution, and participates in inter-organelle transport (Dogterom and Koenderink, 2019). Elevated cytosolic calcium can activate histone deacetylase 6, which deacetylates and depolymerizes α-tubulin, thereby degrading microtubules (Zhang et al., 2014). Animal experiments have demonstrated that histone deacetylase inhibition can reverse cardiac fibroblast activation and improve diastolic dysfunction (Travers et al., 2021). Diastolic dysfunction, which shares risk factors with AF, promotes arrhythmogenesis through mechanisms such as increased atrial afterload, myocardial stretch, and wall stress (Rosenberg and Manning, 2012). The frequent coexistence of AF and diastolic dysfunction suggests shared underlying pathological mechanisms (Packer et al., 2020). Structural cytoskeletal abnormalities impede inter-organelle communication; for instance, impaired microtubule function can disrupt mitochondrial mechanosensing, leading to abnormally high intracellular calcium levels and promoting ectopic excitations (Figure 4) (Miragoli et al., 2016).

#### 3.2.4 Disruption of protein homeostasis in atrial cardiomyocytes

Protein homeostasis is essential for normal physiological function and organismal development and aging; its disruption can lead to various metabolic, neurodegenerative, and cardiovascular diseases (Balch et al., 2008). AF has been associated with abnormalities in the protein quality-control system (Brundel et al., 2022; Brundel et al., 2006a), which comprises chaperone proteins (e.g., heat shock proteins [HSPs]), the ubiquitin–proteasome system, and the autophagy system (Henning and Brundel, 2017).

Rapid atrial excitation can cause myolysis, a process against which HSPs can confer protection (Brundel et al., 2006b). Under physiological conditions, HSPs localize to microtubules and stabilize the cytoskeleton (Hu et al., 2019). At AF onset, HSP27 expression increases in atrial myocytes but gradually depletes with AF prolongation, leading to myocyte dissolution (Brundel et al., 2006a). Therefore, increasing HSP expression may protect atrial cell function and represent a novel strategy to prevent clinical AF (Brundel et al., 2006a; Brundel et al., 2008).

Autophagy, the lysosomal degradation of cellular components (Mizushima and Komatsu, 2011), includes macro-autophagy, micro-autophagy, and chaperone-mediated autophagy (Mizushima and Levine, 2020). AF induces endoplasmic reticulum stress and activates macro-autophagy; however, excessive activation in atrial myocytes promotes atrial remodeling. Conversely, inhibiting intracellular autophagy in both animal and human atria can prevent AF-induced cardiac contractile dysfunction (Wiersma et al., 2017). In summary, disrupted protein homeostasis during cellular senescence contributes to AF development.

#### 3.2.5 Alterations in gap junction proteins of atrial myocytes

**FIGURE 5:**
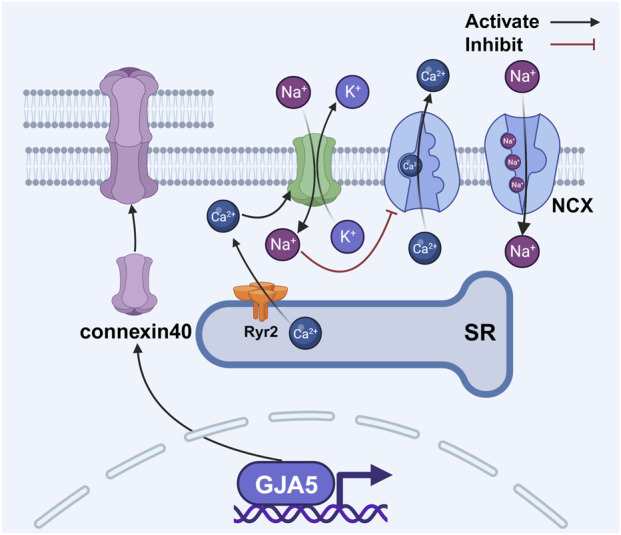
Changes in gap junction proteins between atrial myocytes disrupt the balance of intracellular calcium. The activation of RyR2 results in an elevation of intracellular calcium concentration, thereby facilitating the activation of connexin 43 hemichannels. This activation, albeit indirectly, serves to modulate the activity of the NCX, leading to further augmentation of intracellular calcium levels. Abbreviations: NCX, sodium–calcium exchanger; RyR2, ryanodine receptor 2; SR, sarcoplasmic reticulum. Created with BioRender.com.

Coordinated atrial contraction relies on gap junctions composed of connexin proteins in cardiac myocytes. The atria express connexin43 and connexin40, with the latter being more abundant in the right atrium (Desplantez, 2017; Leybaert et al., 2017; van der Velden and Jongsma, 2002). Structurally, six connexins form a hemichannel, and two hemichannels connect to create a gap junction (Chaldoupi et al., 2009). These junctions enable rapid electrical synchronization between adjacent atrial myocytes (Rohr, 2004), but external stressors can alter them, causing conduction delays (Saffitz, 2006; Yan et al., 2018a) that promote reentrant circuits. Changes in the expression and distribution of connexin40 and connexin43 contribute to atrial conduction heterogeneity (Ryu et al., 2007; Choi et al., 2012). During cellular senescence, gap junction proteins undergo changes, making them a proposed therapeutic target for AF (Spach and Starmer, 1995; Sykora et al., 2023). A large meta-analysis of over 180,000 AF patients implicated cell junction genes such as Plakophilin-2 in AF pathogenesis, suggesting that age-related functional decline of these genes may drive atrial fibrosis and electrical remodeling, ultimately elevating AF risk (Roselli et al., 2025). Supporting this, studies in a mouse model of heart failure revealed decreased connexin43 and connexin40 expression, contributing to abnormal signal conduction (Tong et al., 2022). Mutations in the *GJA5* gene (encoding connexin40) may predispose individuals to idiopathic AF by impairing gap junction assembly or electrical coupling (Gollob et al., 2006). Conversely, animal research demonstrates that gene transfer-mediated overexpression of connexin40 and connexin43 can reduce AF occurrence (Figure 5) (Igarashi et al., 2012).

In addition to gap junctions, connexin hemichannels also play a role in AF. Activation of RyR2 increases intracellular calcium concentration, which promotes the opening of connexin43 hemichannels (Lissoni et al., 2021). These hemichannels function as non-selective pores, allowing potassium efflux and sodium influx. The resulting increase in intracellular sodium levels inhibits the sodium–calcium exchanger (NCX), leading to further calcium accumulation (Fakuade et al., 2021). This elevated calcium facilitates sarcoplasmic reticulum calcium release and delays atrial repolarization, thus collectively increasing atrial excitability and contributing to AF occurrence (Figure 5) (Voigt et al., 2012).

### 3.3 Alterations in cell-cycle-related genes and activation of the ERK signaling pathway

#### 3.3.1 P16 signaling pathway

P16 (cyclin-dependent kinase inhibitor 2A or P16INK4A) regulates the cell cycle by inhibiting the cyclin-dependent kinase (CDK) 4/6 complex. This inhibition prevents retinoblastoma protein (Rb) phosphorylation and the dissociation of the Rb–E2F complex, thereby suppressing E2F transcriptional activity and inducing cell cycle arrest and senescence (Kumari and Jat, 2021). P16 levels increase exponentially with age in various stem cell tissues and mouse models, limiting regenerative capacity and suggesting an acceleration of senescent cell accumulation over time (Molofsky et al., 2006; Krishnamurthy et al., 2006; Janzen et al., 2006; Krishnamurthy et al., 2004; Burd et al., 2013; Herbig et al., 2006; Liu et al., 2009). P16 is also involved in tissue injury repair, with its promoter activating after damage and silencing upon repair (Demaria et al., 2014; Sorrentino et al., 2014). As a key senescence marker alongside p21 (Gasek et al., 2021; Wang et al., 2022), p16 can be induced by ROS and other stressors (Chen et al., 2015). For instance, TGF-β1 increases ROS production to upregulate both p16 and p21, leading to stem cell apoptosis (Fan et al., 2019). While p53 initiates senescence, p16 maintains it (Stein et al., 1999; Alcorta et al., 1996), a cooperation underscored by their shared downstream target, the E2F transcription factor (Rowland et al., 2002). Mechanistically, p16 binding to CDK4/6 disrupts its interaction with cyclin D, blocking cell cycle initiation (Pavletich, 1999). Sustained p16 expression stably inhibits proliferation by maintaining Rb activation and E2F suppression, arresting cells in the G1 phase (Bazarov et al., 2012; Sherr and McCormick, 2002) and influencing senescence-associated heterochromatin foci formation (Narita et al., 2003). Cells expressing p16 undergo prolonged, telomere-length-independent cell cycle arrest, which is characteristic of oncogene-induced senescence (Figure 3) (Gray-Schopfer et al., 2006). Epigenetically, p16 is negatively regulated by histone H3 lysine 27 trimethylation, a modification promoted by the enhancer of zeste homolog 2 (Al-Hasani et al., 2022; Jacobs et al., 1999; Mirzaei et al., 2022).

P16 plays a dual role in both aging and AF. Expression of p53, p16, and tissue factor is significantly increased in the right atrial appendage of AF patients, and since p53 and p16 are markers of cellular senescence, this indicates that AF progression is associated with cellular senescence (Jesel et al., 2019). P16 may promote AF by facilitating atrial fibrosis. Analysis of left atrial appendage tissue from mice with AF shows increased fibrosis in regions positive for senescence-associated beta-galactosidase and p16 (Xie et al., 2017). Research in diabetic mouse models found that accumulation of advanced glycation endproducts increases p16/Rb protein expression, increases the number of senescence-associated β-galactosidase-positive cells, prolongs action potential duration, and increases AF susceptibility. Critically, intervening in the p16/Rb pathway reverses these effects, suggesting that it could be a new therapeutic strategy (Zheng et al., 2022). However, given the differences in electrophysiology (e.g., shorter action potential durations in humans), further human studies are needed to confirm this hypothesis. Supporting the clinical relevance, analysis of intraoperative left atrial appendage tissue from AF patients revealed upregulation of p16, p21, and p53, with p21 identified as an independent predictor of early AF recurrence (Adili et al., 2022). Although numerous studies associate p16 upregulation with AF occurrence, the underlying mechanisms remain unclear.

#### 3.3.2 P21 signaling pathway

P21 (cyclin-dependent kinase inhibitor 1) is a gene involved in aging and cancer that inhibits cyclin-dependent kinase complexes to regulate the cell cycle, DNA replication, and repair (Kumari and Jat, 2021). Located on chromosome 6p21.2, it encodes a protein that, when expressed, induces G1-phase cell cycle arrest (Xiong et al., 1993; Yoshikawa et al., 2010). Its function depends on subcellular localization: nuclear p21 inhibits cell division and acts as a tumor suppressor, whereas cytoplasmic p21 promotes tumor growth (Maria Teresa and Stefania, 2012; Romanov et al., 2012; Romanov and Rudolph, 2016). P21 promotes cellular senescence by interacting with various pathways. For instance, TGF-β1 increases ROS production to upregulate p21 and induce stem cell apoptosis (Fan et al., 2019), and the ERK1/2 pathway can activate p21 to trigger premature senescence (Cammarano et al., 2005). Activated ERK1/2 promotes senescence by modulating proteins such as ribosomal S6 kinase, Sprouty, and c-myc (Campaner et al., 2010; Sun et al., 2018). It also interacts with other pathways, such as promoting SASP production via NF-κB activation (Catanzaro et al., 2014), enhancing mTOR signaling to help sustain senescence (Leontieva and Blagosklonny, 2010; Laberge et al., 2015) and coinciding with the gradual activation of the tumor-suppressor p38 (Kim et al., 2003; Freund et al., 2011). Notably, mTOR signaling regulates SASP, and its inhibition with rapamycin can reduce SASP, thereby alleviating inflammation and senescence (Weichhart, 2018; Wang et al., 2017). Klotho delays aging by inhibiting IGF-1 signaling and reducing ERK1/2 phosphorylation (Dalton et al., 2017; Kurosu et al., 2005; Wolf et al., 2008). SIRT1 can inhibit p53 (Tran et al., 2014), IGF-1 can inhibit SIRT1 (Tran et al., 2014), and Klotho can inhibit the action of IGF-1 (Wolf et al., 2008). Therefore, Klotho can indirectly inhibit the activation of p53, thereby delaying cellular senescence (Figure 3).

Apart from its association with aging, p21 is also linked to AF occurrence. A human study found increased senescent cell area in the left atrial appendage (LAA) of AF patients, with those experiencing recurrence exhibiting higher p21 mRNA levels. Multivariate analysis confirmed p21 as an independent predictor of early AF recurrence (Adili et al., 2022). Further experiments demonstrated that inhibiting p21 reduces tachycardia-pacing-induced cellular senescence, decreases the release of inflammatory factors (IL-1β and IL-6), and partially restores sarcoplasmic reticulum protein expression, suggesting a key role for p21 in AF-related senescence and electrical remodeling (Adili et al., 2022). Although the exact mechanisms are not fully understood, p21 is considered to contribute to AF by regulating atrial remodeling. For instance, thrombin-induced upregulation of p21 and p16 promotes cellular senescence in atrial endothelial cells, leading to atrial remodeling (Hasan et al., 2019; Jansen et al., 2025).

#### 3.3.3 P53 signaling pathway

P53, a tumor suppressor gene, plays a central role in cellular senescence by maintaining genomic integrity. It facilitates DNA repair and induces cell cycle arrest to prevent the proliferation of damaged cells. Upon DNA damage, the p53–p21 and p16–pRb axes are activated, leading to cell cycle arrest and initiating processes for DNA repair, cellular senescence, or apoptosis (Ou and Schumacher, 2018). Consequently, lowered p53 levels can result in genomic instability (Williams and Schumacher, 2016), while premature aging syndromes with defective DNA repair are often characterized by genomic instability and increased p53 activity (Reinhardt and Schumacher, 2012). P53 thus exhibits a dual function, balancing tumor-suppressive and aging-promoting activities (Timofeev et al., 2020). For instance, phosphorylation of its DNA-binding domain can reduce tumor-suppressive activity and prevent senescence, whereas p53 activation can also induce senescence and apoptosis, contributing to organismal degeneration and loss of regenerative capacity (Timofeev et al., 2020; Schumacher et al., 2021; Serrano et al., 1997).

Mitochondrial dysfunction is a key component of cellular senescence (Wiley et al., 2016). P53 can exacerbate this by suppressing peroxisome proliferator-activated receptor gamma coactivator 1-alpha and 1-beta, leading to increased ROS production. This, in turn, activates p53 through a feedback loop (Sahin and DePinho, 2012). Once established, this cycle drives continuous cellular senescence via the p21 and plasminogen activator inhibitor-1 (PAI-1) pathways (Dulic et al., 1994; Kortlever et al., 2006; El-Deiry et al., 1993). Murine double minute 2 protein is the most well-known p53 inhibitor (Haupt et al., 1997; Zhu et al., 2022), although no clinical drugs currently target this interaction (Figure 3).

Interestingly, aside from its role in aging, p53 is also associated with AF occurrence. Bioinformatics analysis of atrial tissue from AF patients revealed differential gene expression, implicating pathways including the “p53 pathway,” thus suggesting its involvement in AF development (Chiang et al., 2015). Supporting this, analysis of right atrial appendage tissue from cardiac surgery patients showed significantly increased expression of p53 and p16 in AF patients, with multivariate analysis identifying them as the sole predictors of AF (Jesel et al., 2019). Furthermore, p53 upregulation was confirmed in the left atrial appendage of AF patients (Adili et al., 2022). These findings consistently demonstrate a close connection between AF and cellular senescence in atrial cells. Mechanistically, studies using HL-1 cell models demonstrated that telomerase reverse transcriptase regulates calcium-handling proteins (SERCA2a, CaV1.2, and NCX1.1) via the p53/PGC-1α pathway. This pathway is aberrantly activated during aging, directly contributing to age-related atrial electrical remodeling and calcium dysregulation. Telomerase reverse transcriptase silencing recapitulated key features of aged cardiomyocytes, including calcium overload, mitochondrial dysfunction, and electrophysiological disturbances—core pathological mechanisms underlying age-related AF (Li et al., 2024).

Studies have indicated a connection between p53 and AF occurrence, though the exact causal relationship and underlying mechanisms remain uncertain. Researchers have identified three genes involved in atrial fibrosis—SERPINE1/PAI-1, TIMP3, and DCN—with PAI-1 being the most significant. Molecular experiments have demonstrated that p53 regulates PAI-1, and this relationship is supported by the increased expression of both p53 and PAI-1, which is observed in atrial tissue from AF patients. These findings suggest that the p53/PAI-1 signaling axis may play a role in the pathophysiology of AF (Li et al., 2021).

## 4 Clinical importance and future directions

This review systematically examines the link between AF and cardiomyocyte senescence. It explores three key mechanisms: inflammatory activation, calcium handling abnormalities, and cell cycle dysregulation. The analysis reveals how aging promotes AF through multiple pathways. Moreover, the review identifies several potential therapeutic targets. These include mitochondrial ROS, NLRP3 inflammasome, and SIRT1/mTOR pathways. These targets serve as both senescence markers and novel intervention points for AF. The findings provide crucial theoretical support for developing anti-aging therapies against AF.

There are several important unanswered questions regarding the role of senescence of atrial myocytes in AF. First, multiple signaling molecules are associated with both AF and cellular senescence, but they are part of complex networks rather than individual pathways. Current research on AF and atrial myocyte senescence is limited by fragmented mechanistic studies, inadequate models, poor cell-type specificity, and challenges in clinical translation. Identifying the key pathway that promotes AF and aging requires further research because interventions targeting these pathways are still under exploration. Second, aging is a complex phenomenon involving intricate alterations in intracellular signaling pathways. Cardiac tissue consists of various cell types, including cardiac myocytes, fibroblasts, conduction bundles, neural tissue, and inflammatory cells. Senescence has distinct effects in each cell type, making it challenging to fully comprehend the relationship between aging and AF. Most studies focus on tissues when investigating AF and aging, leaving uncertainty regarding the specific cell type for which senescence has the greatest impact on AF. The specific cell type that plays a major role in the occurrence of AF awaits clarification. Third, promotion of AF involves multiple cell types in the heart, including fibroblasts and endothelial cells, and senescence of atrial myocytes alone may not be sufficient to cause AF. The relationship between various cell types in the occurrence and maintenance of AF requires further investigation.

## 5 Conclusion

This review proposes that atrial myocyte senescence is a central driver in the pathogenesis of AF, moving beyond the traditional focus on chronological age. The evidence synthesizes into a coherent senescence–AF axis. The interplay of key mechanisms—including mitochondrial dysfunction, NLRP3 inflammasome activation, the SASP, and dysregulation of SIRT1 and mTOR signaling—creates a pro-arrhythmic substrate. This is further compounded by epigenetic alterations, disrupted calcium handling, and the action of cell cycle inhibitors such as p16, p21, and p53, which collectively promote electrical and structural remodeling. These pathways converge to facilitate both the triggers and maintenance of AF. Targeting these fundamental aging mechanisms—such as with SIRT1 activators, NLRP3 inhibitors, or mTOR modulators—holds significant promise for developing novel therapeutic strategies. Future research must delineate the dominant pathways and clarify the contribution of senescence in specific cardiac cell types to translate these insights into effective anti-arrhythmic treatments. Moreover, translating the senescence–AF axis from a concept to clinical practice is a paramount future goal, necessitating interdisciplinary collaboration to advance therapies that directly target cardiac aging into cardiovascular clinical trials.

## References

[B1] Acosta J. C. Banito A. Wuestefeld T. Georgilis A. Janich P. Morton J. P. (2013). A complex secretory program orchestrated by the inflammasome controls paracrine senescence. Nat. Cell Biol. 15, 978–990. 10.1038/ncb2784 23770676 PMC3732483

[B2] Adili A. Zhu X. Cao H. Tang X. Wang Y. Wang J. (2022). Atrial fibrillation underlies cardiomyocyte senescence and contributes to deleterious atrial remodeling during disease progression. Aging Dis. 13, 298–312. 10.14336/AD.2021.0619 35111375 PMC8782549

[B3] Ajoolabady A. Nattel S. Lip G. Y. H. Ren J. (2022). Inflammasome signaling in atrial fibrillation: JACC state-of-the-art review. J. Am. Coll. Cardiol. 79, 2349–2366. 10.1016/j.jacc.2022.03.379 35680186 PMC8972346

[B4] Al-Hasani K. Khurana I. Mariana L. Loudovaris T. Maxwell S. Harikrishnan K. N. (2022). Inhibition of pancreatic EZH2 restores progenitor insulin in T1D donor. Signal Transduct. Target Ther. 7, 248. 10.1038/s41392-022-01034-7 35864094 PMC9304326

[B5] Alcorta D. A. Xiong Y. Phelps D. Hannon G. Beach D. Barrett J. C. (1996). Involvement of the cyclin-dependent kinase inhibitor p16 (INK4a) in replicative senescence of normal human fibroblasts. Proc. Natl. Acad. Sci. U. S. A. 93, 13742–13747. 10.1073/pnas.93.24.13742 8943005 PMC19411

[B6] Allessie M. A. De Groot N. M. Houben R. P. Schotten U. Boersma E. Smeets J. L. (2010). Electropathological substrate of long-standing persistent atrial fibrillation in patients with structural heart disease: longitudinal dissociation. Circ. Arrhythm. Electrophysiol. 3, 606–615. 10.1161/CIRCEP.109.910125 20719881

[B7] Almeida M. Porter R. M. (2019). Sirtuins and FoxOs in osteoporosis and osteoarthritis. Bone 121, 284–292. 10.1016/j.bone.2019.01.018 30738214 PMC6812652

[B8] Anderson R. Lagnado A. Maggiorani D. Walaszczyk A. Dookun E. Chapman J. (2019). Length-independent telomere damage drives post-mitotic cardiomyocyte senescence. EMBO J. 38, e100492. 10.15252/embj.2018100492 30737259 PMC6396144

[B9] Andrade J. Khairy P. Dobrev D. Nattel S. (2014). The clinical profile and pathophysiology of atrial fibrillation: relationships among clinical features, epidemiology, and mechanisms. Circ. Res. 114, 1453–1468. 10.1161/CIRCRESAHA.114.303211 24763464

[B10] Arriola Apelo S. I. Neuman J. C. Baar E. L. Syed F. A. Cummings N. E. Brar H. K. (2016a). Alternative rapamycin treatment regimens mitigate the impact of rapamycin on glucose homeostasis and the immune system. Aging Cell 15, 28–38. 10.1111/acel.12405 26463117 PMC4717280

[B11] Arriola Apelo S. I. Pumper C. P. Baar E. L. Cummings N. E. Lamming D. W. (2016b). Intermittent administration of rapamycin extends the life span of female C57BL/6J mice. J. Gerontol. A Biol. Sci. Med. Sci. 71, 876–881. 10.1093/gerona/glw064 27091134 PMC4906329

[B12] Balch W. E. Morimoto R. I. Dillin A. Kelly J. W. (2008). Adapting proteostasis for disease intervention. Science. 319, 916–919. 10.1126/science.1141448 18276881

[B13] Bandeen-Roche K. Xue Q. L. Ferrucci L. Walston J. Guralnik J. M. Chaves P. (2006). Phenotype of frailty: characterization in the women's health and aging studies. J. Gerontol. A Biol. Sci. Med. Sci. 61, 262–266. 10.1093/gerona/61.3.262 16567375

[B14] Bazarov A. V. Lee W. J. Bazarov I. Bosire M. Hines W. C. Stankovich B. (2012). The specific role of pRb in p16 (INK4A) -mediated arrest of normal and malignant human breast cells. Cell Cycle. 11, 1008–1013. 10.4161/cc.11.5.19492 22333593 PMC3323799

[B15] Bers D. M. (2014). Cardiac sarcoplasmic reticulum calcium leak: basis and roles in cardiac dysfunction. Annu. Rev. Physiol. 76, 107–127. 10.1146/annurev-physiol-020911-153308 24245942

[B16] Birch J. Gil J. (2020). Senescence and the SASP: many therapeutic avenues. Genes. Dev. 34, 1565–1576. 10.1101/gad.343129.120 33262144 PMC7706700

[B17] Bitto A. Ito T. K. Pineda V. V. Letexier N. J. Huang H. Z. Sutlief E. (2016). Transient rapamycin treatment can increase lifespan and healthspan in middle-aged mice. Elife. 5, e16351. 10.7554/eLife.16351 27549339 PMC4996648

[B18] Bjedov I. Toivonen J. M. Kerr F. Slack C. Jacobson J. Foley A. (2010). Mechanisms of life span extension by rapamycin in the fruit fly *Drosophila melanogaster* . Cell Metab. 11, 35–46. 10.1016/j.cmet.2009.11.010 20074526 PMC2824086

[B19] Brown E. J. Albers M. W. Shin T. B. Ichikawa K. Keith C. T. Lane W. S. (1994). A mammalian protein targeted by G1-arresting rapamycin-receptor complex. Nature. 369, 756–758. 10.1038/369756a0 8008069

[B20] Brundel B. J. Henning R. H. Ke L. Van Gelder I. C. Crijns H. J. Kampinga H. H. (2006a). Heat shock protein upregulation protects against pacing-induced myolysis in HL-1 atrial myocytes and in human atrial fibrillation. J. Mol. Cell Cardiol. 41, 555–562. 10.1016/j.yjmcc.2006.06.068 16876820

[B21] Brundel B. J. Shiroshita-Takeshita A. Qi X. Yeh Y. H. Chartier D. Van Gelder I. C. (2006b). Induction of heat shock response protects the heart against atrial fibrillation. Circ. Res. 99, 1394–1402. 10.1161/01.RES.0000252323.83137.fe 17110598

[B22] Brundel B. J. Ke L. Dijkhuis A. J. Qi X. Shiroshita-Takeshita A. Nattel S. (2008). Heat shock proteins as molecular targets for intervention in atrial fibrillation. Cardiovasc Res. 78, 422–428. 10.1093/cvr/cvn060 18326558

[B23] Brundel B. Ai X. Hills M. T. Kuipers M. F. Lip G. Y. H. De Groot N. M. S. (2022). Atrial fibrillation. Nat. Rev. Dis. Prim. 8, 21. 10.1038/s41572-022-00347-9 35393446

[B24] Bugyei-Twum A. Ford C. Civitarese R. Seegobin J. Advani S. L. Desjardins J. F. (2018). Sirtuin 1 activation attenuates cardiac fibrosis in a rodent pressure overload model by modifying Smad2/3 transactivation. Cardiovasc Res. 114, 1629–1641. 10.1093/cvr/cvy131 29800064 PMC6148332

[B25] Burd C. E. Sorrentino J. A. Clark K. S. Darr D. B. Krishnamurthy J. Deal A. M. (2013). Monitoring tumorigenesis and senescence *in vivo* with a p16(INK4a)-luciferase model. Cell. 152, 340–351. 10.1016/j.cell.2012.12.010 23332765 PMC3718011

[B26] Calvanese V. Lara E. Kahn A. Fraga M. F. (2009). The role of epigenetics in aging and age-related diseases. Ageing Res. Rev. 8, 268–276. 10.1016/j.arr.2009.03.004 19716530

[B27] Cammarano M. S. Nekrasova T. Noel B. Minden A. (2005). Pak4 induces premature senescence via a pathway requiring p16INK4/p19ARF and mitogen-activated protein kinase signaling. Mol. Cell Biol. 25, 9532–9542. 10.1128/MCB.25.21.9532-9542.2005 16227603 PMC1265798

[B28] Campaner S. Doni M. Verrecchia A. Faga G. Bianchi L. Amati B. (2010). Myc, Cdk2 and cellular senescence: old players, new game. Cell Cycle. 9, 3655–3661. 10.4161/cc.9.18.13049 20818171

[B29] Campbell H. M. Quick A. P. Abu-Taha I. Chiang D. Y. Kramm C. F. Word T. A. (2020). Loss of SPEG inhibitory phosphorylation of ryanodine receptor Type-2 promotes atrial fibrillation. Circulation. 142, 1159–1172. 10.1161/CIRCULATIONAHA.120.045791 32683896 PMC7508800

[B30] Campisi J. Kapahi P. Lithgow G. J. Melov S. Newman J. C. Verdin E. (2019). From discoveries in ageing research to therapeutics for healthy ageing. Nature. 571, 183–192. 10.1038/s41586-019-1365-2 31292558 PMC7205183

[B31] Catanzaro J. M. Sheshadri N. Pan J. A. Sun Y. Shi C. Li J. (2014). Oncogenic ras induces inflammatory cytokine production by upregulating the squamous cell carcinoma antigens SerpinB3/B4. Nat. Commun. 5, 3729. 10.1038/ncomms4729 24759783 PMC4025922

[B32] Chaldoupi S. M. Loh P. Hauer R. N. De Bakker J. M. Van Rijen H. V. (2009). The role of connexin40 in atrial fibrillation. Cardiovasc Res. 84, 15–23. 10.1093/cvr/cvp203 19535379

[B33] Chapman J. Fielder E. Passos J. F. (2019). Mitochondrial dysfunction and cell senescence: deciphering a complex relationship. FEBS Lett. 593, 1566–1579. 10.1002/1873-3468.13498 31211858

[B34] Chelu M. G. Sarma S. Sood S. Wang S. Van Oort R. J. Skapura D. G. (2009). Calmodulin kinase II-mediated sarcoplasmic reticulum Ca2+ leak promotes atrial fibrillation in mice. J. Clin. Invest. 119, 1940–1951. 10.1172/jci37059 19603549 PMC2701862

[B35] Chen S. A. Hsieh M. H. Tai C. T. Tsai C. F. Prakash V. S. Yu W. C. (1999). Initiation of atrial fibrillation by ectopic beats originating from the pulmonary veins: electrophysiological characteristics, pharmacological responses, and effects of radiofrequency ablation. Circulation. 100, 1879–1886. 10.1161/01.cir.100.18.1879 10545432

[B36] Chen P. M. Lin C. H. Li N. T. Wu Y. M. Lin M. T. Hung S. C. (2015). c-Maf regulates pluripotency genes, proliferation/self-renewal, and lineage commitment in ROS-mediated senescence of human mesenchymal stem cells. Oncotarget. 6, 35404–35418. 10.18632/oncotarget.6178 26496036 PMC4742114

[B37] Chen C. Zhou M. Ge Y. Wang X. (2020). SIRT1 and aging related signaling pathways. Mech. Ageing Dev. 187, 111215. 10.1016/j.mad.2020.111215 32084459

[B38] Chen J. Chen H. Pan L. (2021a). SIRT1 and gynecological malignancies (Review). Oncol. Rep. 45, 43. 10.3892/or.2021.7994 33649834 PMC7934219

[B39] Chen Y. C. Voskoboinik A. Gerche A. L. Marwick T. H. Mcmullen J. R. (2021b). Prevention of pathological atrial remodeling and atrial fibrillation: JACC state-of-the-art review. J. Am. Coll. Cardiol. 77, 2846–2864. 10.1016/j.jacc.2021.04.012 34082914

[B40] Chen Y. C. Wijekoon S. Matsumoto A. Luo J. Kiriazis H. Masterman E. (2024). Distinct functional and molecular profiles between physiological and pathological atrial enlargement offer potential new therapeutic opportunities for atrial fibrillation. Clin. Sci. (Lond). 138, 941–962. 10.1042/CS20240178 39018488 PMC11292366

[B41] Chiang D. Y. Kongchan N. Beavers D. L. Alsina K. M. Voigt N. Neilson J. R. (2014). Loss of microRNA-106b-25 cluster promotes atrial fibrillation by enhancing ryanodine receptor type-2 expression and calcium release. Circ. Arrhythm. Electrophysiol. 7, 1214–1222. 10.1161/CIRCEP.114.001973 25389315 PMC4270890

[B42] Chiang D. Y. Zhang M. Voigt N. Alsina K. M. Jakob H. Martin J. F. (2015). Identification of microRNA-mRNA dysregulations in paroxysmal atrial fibrillation. Int. J. Cardiol. 184, 190–197. 10.1016/j.ijcard.2015.01.075 25706326 PMC4417399

[B43] Choi E. K. Chang P. C. Lee Y. S. Lin S. F. Zhu W. Maruyama M. (2012). Triggered firing and atrial fibrillation in transgenic mice with selective atrial fibrosis induced by overexpression of TGF-beta1. Circ. J. 76, 1354–1362. 10.1253/circj.cj-11-1301 22447020 PMC3593311

[B44] Christ T. Boknik P. Wohrl S. Wettwer E. Graf E. M. Bosch R. F. (2004). L-type Ca2+ current downregulation in chronic human atrial fibrillation is associated with increased activity of protein phosphatases. Circulation. 110, 2651–2657. 10.1161/01.CIR.0000145659.80212.6A 15492323

[B45] Coppe J. P. Patil C. K. Rodier F. Sun Y. Munoz D. P. Goldstein J. (2008). Senescence-associated secretory phenotypes reveal cell-nonautonomous functions of oncogenic RAS and the p53 tumor suppressor. PLoS Biol. 6, 2853–2868. 10.1371/journal.pbio.0060301 19053174 PMC2592359

[B46] Coppe J. P. Desprez P. Y. Krtolica A. Campisi J. (2010). The senescence-associated secretory phenotype: the dark side of tumor suppression. Annu. Rev. Pathol. 5, 99–118. 10.1146/annurev-pathol-121808-102144 20078217 PMC4166495

[B47] Cordero M. D. Williams M. R. Ryffel B. (2018). AMP-activated protein kinase regulation of the NLRP3 inflammasome during aging. Trends Endocrinol. Metab. 29, 8–17. 10.1016/j.tem.2017.10.009 29150317

[B48] Dalton G. D. Xie J. An S. W. Huang C. L. (2017). New insights into the mechanism of action of soluble klotho. Front. Endocrinol. (Lausanne) 8, 323. 10.3389/fendo.2017.00323 29250031 PMC5715364

[B49] Davis R. J. (2000). Signal transduction by the JNK group of MAP kinases. Cell. 103, 239–252. 10.1016/s0092-8674(00)00116-1 11057897

[B50] Day K. Waite L. L. Thalacker-Mercer A. West A. Bamman M. M. Brooks J. D. (2013). Differential DNA methylation with age displays both common and dynamic features across human tissues that are influenced by CpG landscape. Genome Biol. 14, R102. 10.1186/gb-2013-14-9-r102 24034465 PMC4053985

[B51] De Mendoza A. Nguyen T. V. Ford E. Poppe D. Buckberry S. Pflueger J. (2022). Large-scale manipulation of promoter DNA methylation reveals context-specific transcriptional responses and stability. Genome Biol. 23, 163. 10.1186/s13059-022-02728-5 35883107 PMC9316731

[B52] Demaria M. Ohtani N. Youssef S. A. Rodier F. Toussaint W. Mitchell J. R. (2014). An essential role for senescent cells in optimal wound healing through secretion of PDGF-AA. Dev. Cell. 31, 722–733. 10.1016/j.devcel.2014.11.012 25499914 PMC4349629

[B53] Desplantez T. (2017). Cardiac Cx43, Cx40 and Cx45 co-assembling: involvement of connexins epitopes in formation of hemichannels and gap junction channels. BMC Cell Biol. 18, 3. 10.1186/s12860-016-0118-4 28124623 PMC5267329

[B54] Ding M. Feng N. Tang D. Feng J. Li Z. Jia M. (2018). Melatonin prevents Drp1-mediated mitochondrial fission in diabetic hearts through SIRT1-PGC1alpha pathway. J. Pineal Res. 65, e12491. 10.1111/jpi.12491 29575122 PMC6099285

[B55] Dogterom M. Koenderink G. H. (2019). Actin-microtubule crosstalk in cell biology. Nat. Rev. Mol. Cell Biol. 20, 38–54. 10.1038/s41580-018-0067-1 30323238

[B56] Donate Puertas R. Meugnier E. Romestaing C. Rey C. Morel E. Lachuer J. (2017). Atrial fibrillation is associated with hypermethylation in human left atrium, and treatment with decitabine reduces atrial tachyarrhythmias in spontaneously hypertensive rats. Transl. Res. 184, 57–67 e5. 10.1016/j.trsl.2017.03.004 28427903

[B57] Dridi H. Kushnir A. Zalk R. Yuan Q. Melville Z. Marks A. R. (2020). Intracellular calcium leak in heart failure and atrial fibrillation: a unifying mechanism and therapeutic target. Nat. Rev. Cardiol. 17, 732–747. 10.1038/s41569-020-0394-8 32555383 PMC8362847

[B58] Dulic V. Kaufmann W. K. Wilson S. J. Tlsty T. D. Lees E. Harper J. W. (1994). p53-dependent inhibition of cyclin-dependent kinase activities in human fibroblasts during radiation-induced G1 arrest. Cell. 76, 1013–1023. 10.1016/0092-8674(94)90379-4 8137420

[B59] Ebana Y. Sun Y. Yang X. Watanabe T. Makita S. Ozaki K. (2019). Pathway analysis with genome-wide association study (GWAS) data detected the association of atrial fibrillation with the mTOR signaling pathway. Int. J. Cardiol. Heart Vasc. 24, 100383. 10.1016/j.ijcha.2019.100383 31321287 PMC6612921

[B60] Eggert T. Wolter K. Ji J. Ma C. Yevsa T. Klotz S. (2016). Distinct functions of senescence-associated immune responses in liver tumor surveillance and tumor progression. Cancer Cell. 30, 533–547. 10.1016/j.ccell.2016.09.003 27728804 PMC7789819

[B61] El-Deiry W. S. Tokino T. Velculescu V. E. Levy D. B. Parsons R. Trent J. M. (1993). WAF1, a potential mediator of p53 tumor suppression. Cell. 75, 817–825. 10.1016/0092-8674(93)90500-p 8242752

[B62] Faget D. V. Ren Q. Stewart S. A. (2019). Unmasking senescence: context-dependent effects of SASP in cancer. Nat. Rev. Cancer. 19, 439–453. 10.1038/s41568-019-0156-2 31235879

[B63] Fakuade F. E. Tomsits P. Voigt N. (2021). Connexin hemichannels in atrial fibrillation: orphaned and irrelevant? Cardiovasc Res. 117, 4–6. 10.1093/cvr/cvaa308 33112373

[B64] Fan C. Ji Q. Zhang C. Xu S. Sun H. Li Z. (2019). TGF-beta induces periodontal ligament stem cell senescence through increase of ROS production. Mol. Med. Rep. 20, 3123–3130. 10.3892/mmr.2019.10580 31432132 PMC6755147

[B65] Feng D. Xu D. Murakoshi N. Tajiri K. Qin R. Yonebayashi S. (2020). Nicotinamide phosphoribosyltransferase (Nampt)/Nicotinamide adenine dinucleotide (NAD) axis suppresses atrial fibrillation by modulating the calcium handling pathway. Int. J. Mol. Sci. 21, 4655. 10.3390/ijms21134655 32629939 PMC7370160

[B66] Ferrucci L. Fabbri E. (2018). Inflammageing: chronic inflammation in ageing, cardiovascular disease, and frailty. Nat. Rev. Cardiol. 15, 505–522. 10.1038/s41569-018-0064-2 30065258 PMC6146930

[B67] Franceschi C. Bonafe M. Valensin S. Olivieri F. De Luca M. Ottaviani E. (2000). Inflamm-aging. An evolutionary perspective on immunosenescence. Ann. N. Y. Acad. Sci. 908, 244–254. 10.1111/j.1749-6632.2000.tb06651.x 10911963

[B68] Freund A. Patil C. K. Campisi J. (2011). p38MAPK is a novel DNA damage response-independent regulator of the senescence-associated secretory phenotype. EMBO J. 30, 1536–1548. 10.1038/emboj.2011.69 21399611 PMC3102277

[B69] Gasek N. S. Kuchel G. A. Kirkland J. L. Xu M. (2021). Strategies for targeting senescent cells in human disease. Nat. Aging. 1, 870–879. 10.1038/s43587-021-00121-8 34841261 PMC8612694

[B70] Gluck S. Guey B. Gulen M. F. Wolter K. Kang T. W. Schmacke N. A. (2017). Innate immune sensing of cytosolic chromatin fragments through cGAS promotes senescence. Nat. Cell Biol. 19, 1061–1070. 10.1038/ncb3586 28759028 PMC5826565

[B71] Gollob M. H. Jones D. L. Krahn A. D. Danis L. Gong X. Q. Shao Q. (2006). Somatic mutations in the connexin 40 gene (GJA5) in atrial fibrillation. N. Engl. J. Med. 354, 2677–2688. 10.1056/NEJMoa052800 16790700

[B72] Gorgoulis V. Adams P. D. Alimonti A. Bennett D. C. Bischof O. Bishop C. (2019). Cellular senescence: defining a path forward. Cell. 179, 813–827. 10.1016/j.cell.2019.10.005 31675495

[B73] Gray-Schopfer V. C. Cheong S. C. Chong H. Chow J. Moss T. Abdel-Malek Z. A. (2006). Cellular senescence in naevi and immortalisation in melanoma: a role for p16? Br. J. Cancer. 95, 496–505. 10.1038/sj.bjc.6603283 16880792 PMC2360676

[B74] Griveau A. Wiel C. Ziegler D. V. Bergo M. O. Bernard D. (2020). The JAK1/2 inhibitor ruxolitinib delays premature aging phenotypes. Aging Cell. 19, e13122. 10.1111/acel.13122 32196928 PMC7189991

[B75] Hahn A. Zuryn S. (2019). Mitochondrial genome (mtDNA) mutations that generate reactive oxygen species. Antioxidants (Basel). 8, 392. 10.3390/antiox8090392 31514455 PMC6769445

[B76] Haigis M. C. Sinclair D. A. (2010). Mammalian sirtuins: biological insights and disease relevance. Annu. Rev. Pathol. 5, 253–295. 10.1146/annurev.pathol.4.110807.092250 20078221 PMC2866163

[B77] Haissaguerre M. Jais P. Shah D. C. Takahashi A. Hocini M. Quiniou G. (1998). Spontaneous initiation of atrial fibrillation by ectopic beats originating in the pulmonary veins. N. Engl. J. Med. 339, 659–666. 10.1056/NEJM199809033391003 9725923

[B78] Han L. Tang Y. Li S. Wu Y. Chen X. Wu Q. (2020a). Protective mechanism of SIRT1 on Hcy-induced atrial fibrosis mediated by TRPC3. J. Cell Mol. Med. 24, 488–510. 10.1111/jcmm.14757 31680473 PMC6933351

[B79] Han Y. Sun W. Ren D. Zhang J. He Z. Fedorova J. (2020b). SIRT1 agonism modulates cardiac NLRP3 inflammasome through pyruvate dehydrogenase during ischemia and reperfusion. Redox Biol. 34, 101538. 10.1016/j.redox.2020.101538 32325423 PMC7176991

[B80] Hannum G. Guinney J. Zhao L. Zhang L. Hughes G. Sadda S. (2013). Genome-wide methylation profiles reveal quantitative views of human aging rates. Mol. Cell. 49, 359–367. 10.1016/j.molcel.2012.10.016 23177740 PMC3780611

[B81] Hansen M. Rubinsztein D. C. Walker D. W. (2018). Autophagy as a promoter of longevity: insights from model organisms. Nat. Rev. Mol. Cell Biol. 19, 579–593. 10.1038/s41580-018-0033-y 30006559 PMC6424591

[B82] Hasan H. Park S. H. Auger C. Belcastro E. Matsushita K. Marchandot B. (2019). Thrombin induces angiotensin II-Mediated senescence in atrial endothelial cells: impact on pro-remodeling patterns. J. Clin. Med. 8, 1570. 10.3390/jcm8101570 31581517 PMC6833093

[B83] Haupt Y. Maya R. Kazaz A. Oren M. (1997). Mdm2 promotes the rapid degradation of p53. Nature. 387, 296–299. 10.1038/387296a0 9153395

[B84] He S. Sharpless N. E. (2017). Senescence in health and disease. Cell. 169, 1000–1011. 10.1016/j.cell.2017.05.015 28575665 PMC5643029

[B85] Heijman J. Muna A. P. Veleva T. Molina C. E. Sutanto H. Tekook M. (2020). Atrial myocyte NLRP3/CaMKII nexus forms a substrate for postoperative atrial fibrillation. Circ. Res. 127, 1036–1055. 10.1161/CIRCRESAHA.120.316710 32762493 PMC7604886

[B86] Henning R. H. Brundel B. (2017). Proteostasis in cardiac health and disease. Nat. Rev. Cardiol. 14, 637–653. 10.1038/nrcardio.2017.89 28660894

[B87] Herbig U. Ferreira M. Condel L. Carey D. Sedivy J. M. (2006). Cellular senescence in aging primates. Science 311, 1257. 10.1126/science.1122446 16456035

[B88] Herranz N. Gallage S. Mellone M. Wuestefeld T. Klotz S. Hanley C. J. (2015). mTOR regulates MAPKAPK2 translation to control the senescence-associated secretory phenotype. Nat. Cell Biol. 17, 1205–1217. 10.1038/ncb3225 26280535 PMC4589897

[B89] Howitz K. T. Bitterman K. J. Cohen H. Y. Lamming D. W. Lavu S. Wood J. G. (2003). Small molecule activators of sirtuins extend *Saccharomyces cerevisiae* lifespan. Nature. 425, 191–196. 10.1038/nature01960 12939617

[B90] Hu X. Li J. Van Marion D. M. S. Zhang D. Brundel B. (2019). Heat shock protein inducer GGA*-59 reverses contractile and structural remodeling via restoration of the microtubule network in experimental atrial fibrillation. J. Mol. Cell Cardiol. 134, 86–97. 10.1016/j.yjmcc.2019.07.006 31302117

[B91] Igarashi T. Finet J. E. Takeuchi A. Fujino Y. Strom M. Greener I. D. (2012). Connexin gene transfer preserves conduction velocity and prevents atrial fibrillation. Circulation. 125, 216–225. 10.1161/CIRCULATIONAHA.111.053272 22158756 PMC3260348

[B92] Ikeda Y. Sato K. Pimentel D. R. Sam F. Shaw R. J. Dyck J. R. (2009). Cardiac-specific deletion of LKB1 leads to hypertrophy and dysfunction. J. Biol. Chem. 284, 35839–35849. 10.1074/jbc.M109.057273 19828446 PMC2791013

[B93] Imai S. Guarente L. (2014). NAD+ and sirtuins in aging and disease. Trends Cell Biol. 24, 464–471. 10.1016/j.tcb.2014.04.002 24786309 PMC4112140

[B94] Imai S. Armstrong C. M. Kaeberlein M. Guarente L. (2000). Transcriptional silencing and longevity protein Sir2 is an NAD-dependent histone deacetylase. Nature. 403, 795–800. 10.1038/35001622 10693811

[B95] Jacobs J. J. Kieboom K. Marino S. Depinho R. A. Van Lohuizen M. (1999). The oncogene and Polycomb-group gene bmi-1 regulates cell proliferation and senescence through the ink4a locus. Nature. 397, 164–168. 10.1038/16476 9923679

[B96] Jansen H. J. Mcrae M. D. Belke D. D. Rose R. A. (2025). Chronic angiotensin-converting enzyme inhibition attenuates frailty and protects against atrial fibrillation in aging mice. Heart rhythm. 22, 452–465. 10.1016/j.hrthm.2024.07.016 39019387

[B97] Janzen V. Forkert R. Fleming H. E. Saito Y. Waring M. T. Dombkowski D. M. (2006). Stem-cell ageing modified by the cyclin-dependent kinase inhibitor p16INK4a. Nature. 443, 421–426. 10.1038/nature05159 16957735

[B98] Jenwitheesuk A. Park S. Wongchitrat P. Tocharus J. Mukda S. Shimokawa I. (2018). Comparing the effects of melatonin with caloric restriction in the hippocampus of aging mice: involvement of Sirtuin1 and the FOXOs pathway. Neurochem. Res. 43, 153–161. 10.1007/s11064-017-2369-7 28770437

[B99] Jesel L. Abbas M. Park S. H. Matsushita K. Kindo M. Hasan H. (2019). Atrial fibrillation progression is associated with cell senescence burden as determined by p53 and p16 expression. J. Clin. Med. 9, 36. 10.3390/jcm9010036 31878008 PMC7019631

[B100] Jin X. Zhang Y. Zhou Y. Luo Y. Han X. Gao Y. (2024). Sirt1 deficiency promotes age-related AF through enhancing atrial necroptosis by activation of RIPK1 acetylation. Circulation Arrhythmia Electrophysiol. 17, e012452. 10.1161/circep.123.012452 39012929

[B101] Kao Y. H. Chen Y. C. Cheng C. C. Lee T. I. Chen Y. J. Chen S. A. (2010). Tumor necrosis factor-alpha decreases sarcoplasmic reticulum Ca2+-ATPase expressions via the promoter methylation in cardiomyocytes. Crit. Care Med. 38, 217–222. 10.1097/CCM.0b013e3181b4a854 19730253

[B102] Katritsis D. Merchant F. M. Mela T. Singh J. P. Heist E. K. Armoundas A. A. (2010). Catheter ablation of atrial fibrillation the search for substrate-driven end points. J. Am. Coll. Cardiol. 55, 2293–2298. 10.1016/j.jacc.2010.03.016 20488298

[B103] Kauppila T. E. S. Kauppila J. H. K. Larsson N. G. (2017). Mammalian mitochondria and aging: an update. Cell Metab. 25, 57–71. 10.1016/j.cmet.2016.09.017 28094012

[B104] Kauppinen A. Suuronen T. Ojala J. Kaarniranta K. Salminen A. (2013). Antagonistic crosstalk between NF-kappaB and SIRT1 in the regulation of inflammation and metabolic disorders. Cell Signal. 25, 1939–1948. 10.1016/j.cellsig.2013.06.007 23770291

[B105] Keith C. T. Schreiber S. L. (1995). PIK-related kinases: DNA repair, recombination, and cell cycle checkpoints. Science. 270, 50–51. 10.1126/science.270.5233.50 7569949

[B106] Kelley N. Jeltema D. Duan Y. He Y. (2019). The NLRP3 inflammasome: an overview of mechanisms of activation and regulation. Int. J. Mol. Sci. 20, 3328. 10.3390/ijms20133328 31284572 PMC6651423

[B107] Kim H. S. Song M. C. Kwak I. H. Park T. J. Lim I. K. (2003). Constitutive induction of p-Erk1/2 accompanied by reduced activities of protein phosphatases 1 and 2A and MKP3 due to reactive oxygen species during cellular senescence. J. Biol. Chem. 278, 37497–37510. 10.1074/jbc.M211739200 12840032

[B108] Kim Y. M. Guzik T. J. Zhang Y. H. Zhang M. H. Kattach H. Ratnatunga C. (2005). A myocardial Nox2 containing NAD(P)H oxidase contributes to oxidative stress in human atrial fibrillation. Circ. Res. 97, 629–636. 10.1161/01.RES.0000183735.09871.61 16123335

[B109] Kortlever R. M. Higgins P. J. Bernards R. (2006). Plasminogen activator inhibitor-1 is a critical downstream target of p53 in the induction of replicative senescence. Nat. Cell Biol. 8, 877–884. 10.1038/ncb1448 16862142 PMC2954492

[B110] Krishnamurthy J. Torrice C. Ramsey M. R. Kovalev G. I. Al-Regaiey K. Su L. (2004). Ink4a/Arf expression is a biomarker of aging. J. Clin. Invest. 114, 1299–1307. 10.1172/JCI22475 15520862 PMC524230

[B111] Krishnamurthy J. Ramsey M. R. Ligon K. L. Torrice C. Koh A. Bonner-Weir S. (2006). p16INK4a induces an age-dependent decline in islet regenerative potential. Nature. 443, 453–457. 10.1038/nature05092 16957737

[B112] Krizhanovsky V. Yon M. Dickins R. A. Hearn S. Simon J. Miething C. (2008). Senescence of activated stellate cells limits liver fibrosis. Cell. 134, 657–667. 10.1016/j.cell.2008.06.049 18724938 PMC3073300

[B113] Kumari R. Jat P. (2021). Mechanisms of cellular senescence: cell cycle arrest and senescence associated secretory phenotype. Front. Cell Dev. Biol. 9, 645593. 10.3389/fcell.2021.645593 33855023 PMC8039141

[B114] Kurosu H. Yamamoto M. Clark J. D. Pastor J. V. Nandi A. Gurnani P. (2005). Suppression of aging in mice by the hormone klotho. Science. 309, 1829–1833. 10.1126/science.1112766 16123266 PMC2536606

[B115] Laberge R. M. Sun Y. Orjalo A. V. Patil C. K. Freund A. Zhou L. (2015). MTOR regulates the pro-tumorigenic senescence-associated secretory phenotype by promoting IL1A translation. Nat. Cell Biol. 17, 1049–1061. 10.1038/ncb3195 26147250 PMC4691706

[B116] Lamkanfi M. Dixit V. M. (2014). Mechanisms and functions of inflammasomes. Cell. 157, 1013–1022. 10.1016/j.cell.2014.04.007 24855941

[B117] Lee H. Y. Lee S. D. Shin K. J. (2016). Forensic DNA methylation profiling from evidence material for investigative leads. BMB Rep. 49, 359–369. 10.5483/bmbrep.2016.49.7.070 27099236 PMC5032003

[B118] Leontieva O. V. Blagosklonny M. V. (2010). DNA damaging agents and p53 do not cause senescence in quiescent cells, while consecutive re-activation of mTOR is associated with conversion to senescence. Aging (Albany NY). 2, 924–935. 10.18632/aging.100265 21212465 PMC3034181

[B119] Leybaert L. Lampe P. D. Dhein S. Kwak B. R. Ferdinandy P. Beyer E. C. (2017). Connexins in cardiovascular and neurovascular health and disease: pharmacological implications. Pharmacol. Rev. 69, 396–478. 10.1124/pr.115.012062 28931622 PMC5612248

[B120] Li N. Chiang D. Y. Wang S. Wang Q. Sun L. Voigt N. (2014). Ryanodine receptor-mediated calcium leak drives progressive development of an atrial fibrillation substrate in a transgenic mouse model. Circulation. 129, 1276–1285. 10.1161/CIRCULATIONAHA.113.006611 24398018 PMC4026172

[B121] Li Q. Lai Y. Gao X. Li X. Deng C. Y. Guo H. (2021). Involvement of plasminogen activator inhibitor-1 and its related molecules in atrial fibrosis in patients with atrial fibrillation. PeerJ. 9, e11488. 10.7717/peerj.11488 34141473 PMC8179226

[B122] Li Y. Xue Q. Liu N. Wang X. Gao S. Chen Y. (2024). Telomerase reverse transcriptase regulates intracellular Ca2+ homeostasis and mitochondrial function via the p53/PGC-1α pathway in HL-1 cells. Front. Bioscience-Landmark. 29, 263. 10.31083/j.fbl2907263 39082363

[B123] Liang Y. Liang B. Chen W. Wu X. R. Liu-Huo W. S. Zhao L. Z. (2021). Potential mechanism of dingji fumai decoction against atrial fibrillation based on network pharmacology, molecular docking, and experimental verification integration strategy. Front. Cardiovasc Med. 8, 712398. 10.3389/fcvm.2021.712398 34859062 PMC8631917

[B124] Liberale L. Montecucco F. Tardif J. C. Libby P. Camici G. G. (2020). Inflamm-ageing: the role of inflammation in age-dependent cardiovascular disease. Eur. Heart J. 41, 2974–2982. 10.1093/eurheartj/ehz961 32006431 PMC7453832

[B125] Lissoni A. Hulpiau P. Martins-Marques T. Wang N. Bultynck G. Schulz R. (2021). RyR2 regulates Cx43 hemichannel intracellular Ca2+-dependent activation in cardiomyocytes. Cardiovasc Res. 117, 123–136. 10.1093/cvr/cvz340 31841141

[B126] Liu Y. Sanoff H. K. Cho H. Burd C. E. Torrice C. Ibrahim J. G. (2009). Expression of p16(INK4a) in peripheral blood T-cells is a biomarker of human aging. Aging Cell. 8, 439–448. 10.1111/j.1474-9726.2009.00489.x 19485966 PMC2752333

[B127] Liu G. Z. Hou T. T. Yuan Y. Hang P. Z. Zhao J. J. Sun L. (2016). Fenofibrate inhibits atrial metabolic remodelling in atrial fibrillation through PPAR-alpha/sirtuin 1/PGC-1alpha pathway. Br. J. Pharmacol. 173, 1095–1109. 10.1111/bph.13438 26787506 PMC5341245

[B128] Liu P. Sun H. Zhou X. Wang Q. Gao F. Fu Y. (2021). CXCL12/CXCR4 axis as a key mediator in atrial fibrillation via bioinformatics analysis and functional identification. Cell Death Dis. 12, 813. 10.1038/s41419-021-04109-5 34453039 PMC8397768

[B129] Lopes-Paciencia S. Saint-Germain E. Rowell M. C. Ruiz A. F. Kalegari P. Ferbeyre G. (2019). The senescence-associated secretory phenotype and its regulation. Cytokine. 117, 15–22. 10.1016/j.cyto.2019.01.013 30776684

[B130] Lujambio A. Akkari L. Simon J. Grace D. Tschaharganeh D. F. Bolden J. E. (2013). Non-cell-autonomous tumor suppression by p53. Cell. 153, 449–460. 10.1016/j.cell.2013.03.020 23562644 PMC3702034

[B131] Luo G. Jian Z. Zhu Y. Zhu Y. Chen B. Ma R. (2019). Sirt1 promotes autophagy and inhibits apoptosis to protect cardiomyocytes from hypoxic stress. Int. J. Mol. Med. 43, 2033–2043. 10.3892/ijmm.2019.4125 30864731 PMC6443335

[B132] Ma J. Chen Q. Ma S. (2021). Left atrial fibrosis in atrial fibrillation: mechanisms, clinical evaluation and management. J. Cell. Mol. Med. 25, 2764–2775. 10.1111/jcmm.16350 33576189 PMC7957273

[B133] Mannick J. B. Morris M. Hockey H. P. Roma G. Beibel M. Kulmatycki K. (2018). TORC1 inhibition enhances immune function and reduces infections in the elderly. Sci. Transl. Med. 10, eaaq1564. 10.1126/scitranslmed.aaq1564 29997249

[B134] Mao Z. Ke Z. Gorbunova V. Seluanov A. (2012). Replicatively senescent cells are arrested in G1 and G2 phases. Aging (Albany NY). 4, 431–435. 10.18632/aging.100467 22745179 PMC3409679

[B135] Maria Teresa P. Stefania C. (2012). The dual role played by p21 may influence the apoptotic or anti-apoptotic fate in cancer. J. Cancer Res. Updat. 1, 189–202. 10.6000/1929-2279.2012.01.02.5

[B136] Marin-Aguilar F. Lechuga-Vieco A. V. Alcocer-Gomez E. Castejon-Vega B. Lucas J. Garrido C. (2020). NLRP3 inflammasome suppression improves longevity and prevents cardiac aging in Male mice. Aging Cell. 19, e13050. 10.1111/acel.13050 31625260 PMC6974709

[B137] Masters S. L. Dunne A. Subramanian S. L. Hull R. L. Tannahill G. M. Sharp F. A. (2010). Activation of the NLRP3 inflammasome by islet amyloid polypeptide provides a mechanism for enhanced IL-1beta in type 2 diabetes. Nat. Immunol. 11, 897–904. 10.1038/ni.1935 20835230 PMC3103663

[B138] Miragoli M. Sanchez-Alonso J. L. Bhargava A. Wright P. T. Sikkel M. Schobesberger S. (2016). Microtubule-dependent mitochondria alignment regulates calcium release in response to nanomechanical stimulus in heart myocytes. Cell Rep. 14, 140–151. 10.1016/j.celrep.2015.12.014 26725114 PMC4983655

[B139] Mirzaei S. Gholami M. H. Hushmandi K. Hashemi F. Zabolian A. Canadas I. (2022). The long and short non-coding RNAs modulating EZH2 signaling in cancer. J. Hematol. Oncol. 15, 18. 10.1186/s13045-022-01235-1 35236381 PMC8892735

[B140] Mitchell S. J. Martin-Montalvo A. Mercken E. M. Palacios H. H. Ward T. M. Abulwerdi G. (2014). The SIRT1 activator SRT1720 extends lifespan and improves health of mice fed a standard diet. Cell Rep. 6, 836–843. 10.1016/j.celrep.2014.01.031 24582957 PMC4010117

[B141] Mizushima N. Komatsu M. (2011). Autophagy: renovation of cells and tissues. Cell. 147, 728–741. 10.1016/j.cell.2011.10.026 22078875

[B142] Mizushima N. Levine B. (2020). Autophagy in human diseases. N. Engl. J. Med. 383, 1564–1576. 10.1056/NEJMra2022774 33053285

[B143] Molofsky A. V. Slutsky S. G. Joseph N. M. He S. Pardal R. Krishnamurthy J. (2006). Increasing p16INK4a expression decreases forebrain progenitors and neurogenesis during ageing. Nature. 443, 448–452. 10.1038/nature05091 16957738 PMC2586960

[B144] Movassagh M. Choy M. K. Knowles D. A. Cordeddu L. Haider S. Down T. (2011). Distinct epigenomic features in end-stage failing human hearts. Circulation. 124, 2411–2422. 10.1161/CIRCULATIONAHA.111.040071 22025602 PMC3634158

[B145] Nacarelli T. Azar A. Sell C. (2016). Mitochondrial stress induces cellular senescence in an mTORC1-dependent manner. Free Radic. Biol. Med. 95, 133–154. 10.1016/j.freeradbiomed.2016.03.008 27016071

[B146] Narita M. Nunez S. Heard E. Narita M. Lin A. W. Hearn S. A. (2003). Rb-mediated heterochromatin formation and silencing of E2F target genes during cellular senescence. Cell. 113, 703–716. 10.1016/s0092-8674(03)00401-x 12809602

[B147] Nattel S. Maguy A. Le Bouter S. Yeh Y. H. (2007). Arrhythmogenic ion-channel remodeling in the heart: heart failure, myocardial infarction, and atrial fibrillation. Physiol. Rev. 87, 425–456. 10.1152/physrev.00014.2006 17429037

[B148] Nattel S. Xiong F. Aguilar M. (2017). Demystifying rotors and their place in clinical translation of atrial fibrillation mechanisms. Nat. Rev. Cardiol. 14, 509–520. 10.1038/nrcardio.2017.37 28383023

[B149] Nattel S. Heijman J. Zhou L. Dobrev D. (2020). Molecular basis of atrial fibrillation pathophysiology and therapy: a translational perspective. Circ. Res. 127, 51–72. 10.1161/CIRCRESAHA.120.316363 32717172 PMC7398486

[B150] Neef S. Dybkova N. Sossalla S. Ort K. R. Fluschnik N. Neumann K. (2010). CaMKII-dependent diastolic SR Ca2+ leak and elevated diastolic Ca2+ levels in right atrial myocardium of patients with atrial fibrillation. Circ. Res. 106, 1134–1144. 10.1161/CIRCRESAHA.109.203836 20056922

[B151] Nopparat C. Sinjanakhom P. Govitrapong P. (2017). Melatonin reverses H(2) O(2) -induced senescence in SH-SY5Y cells by enhancing autophagy via sirtuin 1 deacetylation of the RelA/p65 subunit of NF-kappaB. J. Pineal Res. 63, e12407. 10.1111/jpi.12407 28295567

[B152] Ou H. L. Schumacher B. (2018). DNA damage responses and p53 in the aging process. Blood 131, 488–495. 10.1182/blood-2017-07-746396 29141944 PMC6839964

[B153] Packer M. Lam C. S. P. Lund L. H. Redfield M. M. (2020). Interdependence of atrial fibrillation and heart failure with a preserved ejection fraction reflects a common underlying atrial and ventricular myopathy. Circulation. 141, 4–6. 10.1161/circulationaha.119.042996 31887078

[B154] Park S. K. Seong R. K. Kim J. A. Son S. J. Kim Y. Yokozawa T. (2016). Oligonol promotes anti-aging pathways via modulation of SIRT1-AMPK-Autophagy pathway. Nutr. Res. Pract. 10, 3–10. 10.4162/nrp.2016.10.1.3 26865910 PMC4742308

[B155] Pastori D. Carnevale R. Nocella C. Novo M. Santulli M. Cammisotto V. (2017). Gut-derived serum lipopolysaccharide is associated with enhanced risk of major adverse cardiovascular events in atrial fibrillation: effect of adherence to mediterranean diet. J. Am. Heart Assoc. 6, e005784. 10.1161/JAHA.117.005784 28584074 PMC5669181

[B156] Pavletich N. P. (1999). Mechanisms of cyclin-dependent kinase regulation: structures of Cdks, their cyclin activators, and Cip and INK4 inhibitors. J. Mol. Biol. 287, 821–828. 10.1006/jmbi.1999.2640 10222191

[B157] Picca A. Mankowski R. T. Burman J. L. Donisi L. Kim J. S. Marzetti E. (2018). Mitochondrial quality control mechanisms as molecular targets in cardiac ageing. Nat. Rev. Cardiol. 15, 543–554. 10.1038/s41569-018-0059-z 30042431 PMC6283278

[B158] Price N. L. Gomes A. P. Ling A. J. Duarte F. V. Martin-Montalvo A. North B. J. (2012). SIRT1 is required for AMPK activation and the beneficial effects of resveratrol on mitochondrial function. Cell Metab. 15, 675–690. 10.1016/j.cmet.2012.04.003 22560220 PMC3545644

[B159] Quintanilla J. G. Shpun S. Jalife J. Filgueiras-Rama D. (2021). Novel approaches to mechanism-based atrial fibrillation ablation. Cardiovasc Res. 117, 1662–1681. 10.1093/cvr/cvab108 33744913 PMC8208747

[B160] Ramis M. R. Esteban S. Miralles A. Tan D. X. Reiter R. J. (2015). Caloric restriction, resveratrol and melatonin: role of SIRT1 and implications for aging and related-diseases. Mech. Ageing Dev. 146-148, 28–41. 10.1016/j.mad.2015.03.008 25824609

[B161] Ramos K. S. Brundel B. (2020). DNA damage, an innocent bystander in atrial fibrillation and other cardiovascular diseases? Front. Cardiovasc Med. 7, 67. 10.3389/fcvm.2020.00067 32411727 PMC7198718

[B162] Ramos-MondragóN R. Lozhkin A. Vendrov A. E. Runge M. S. Isom L. L. Madamanchi N. R. (2023). NADPH oxidases and oxidative stress in the pathogenesis of atrial fibrillation. Antioxidants. 12, 1833. 10.3390/antiox12101833 37891912 PMC10604902

[B163] Reinhardt H. C. Schumacher B. (2012). The p53 network: cellular and systemic DNA damage responses in aging and cancer. Trends Genet. 28, 128–136. 10.1016/j.tig.2011.12.002 22265392 PMC4120491

[B164] Robida-Stubbs S. Glover-Cutter K. Lamming D. W. Mizunuma M. Narasimhan S. D. Neumann-Haefelin E. (2012). TOR signaling and rapamycin influence longevity by regulating SKN-1/Nrf and DAF-16/FoxO. Cell Metab. 15, 713–724. 10.1016/j.cmet.2012.04.007 22560223 PMC3348514

[B165] Rohr S. (2004). Role of gap junctions in the propagation of the cardiac action potential. Cardiovasc Res. 62, 309–322. 10.1016/j.cardiores.2003.11.035 15094351

[B166] Romanov V. S. Rudolph K. L. (2016). p21 shapes cancer evolution. Nat. Cell Biol. 18, 722–724. 10.1038/ncb3382 27350444

[B167] Romanov V. S. Pospelov V. A. Pospelova T. V. (2012). Cyclin-dependent kinase inhibitor p21(Waf1): contemporary view on its role in senescence and oncogenesis. Biochem. (Mosc). 77, 575–584. 10.1134/S000629791206003X 22817456

[B168] Roselli C. Surakka I. Olesen M. S. Sveinbjornsson G. Marston N. A. Choi S. H. (2025). Meta-analysis of genome-wide associations and polygenic risk prediction for atrial fibrillation in more than 180,000 cases. Nat. Genet. 57, 539–547. 10.1038/s41588-024-02072-3 40050429 PMC12094172

[B169] Rosenberg M. A. Manning W. J. (2012). Diastolic dysfunction and risk of atrial fibrillation. Circulation. 126, 2353–2362. 10.1161/circulationaha.112.113233 23129702

[B170] Rowland B. D. Denissov S. G. Douma S. Stunnenberg H. G. Bernards R. Peeper D. S. (2002). E2F transcriptional repressor complexes are critical downstream targets of p19(ARF)/p53-induced proliferative arrest. Cancer Cell. 2, 55–65. 10.1016/s1535-6108(02)00085-5 12150825

[B171] Ryu K. Li L. Khrestian C. M. Matsumoto N. Sahadevan J. Ruehr M. L. (2007). Effects of sterile pericarditis on connexins 40 and 43 in the atria: correlation with abnormal conduction and atrial arrhythmias. Am. J. Physiol. Heart Circ. Physiol. 293, H1231–H1241. 10.1152/ajpheart.00607.2006 17434983

[B172] Ryu J. K. Kim S. J. Rah S. H. Kang J. I. Jung H. E. Lee D. (2017). Reconstruction of LPS transfer Cascade reveals structural determinants within LBP, CD14, and TLR4-MD2 for efficient LPS recognition and transfer. Immunity. 46, 38–50. 10.1016/j.immuni.2016.11.007 27986454

[B173] Sabatini D. M. Erdjument-Bromage H. Lui M. Tempst P. Snyder S. H. (1994). RAFT1: a mammalian protein that binds to FKBP12 in a rapamycin-dependent fashion and is homologous to yeast TORs. Cell. 78, 35–43. 10.1016/0092-8674(94)90570-3 7518356

[B174] Saffitz J. E. (2006). Connexins, conduction, and atrial fibrillation. N. Engl. J. Med. 354, 2712–2714. 10.1056/NEJMe068088 16790707

[B175] Sahin E. Depinho R. A. (2012). Axis of ageing: telomeres, p53 and mitochondria. Nat. Rev. Mol. Cell Biol. 13, 397–404. 10.1038/nrm3352 22588366 PMC3718675

[B176] Schumacher B. Pothof J. Vijg J. Hoeijmakers J. H. J. (2021). The central role of DNA damage in the ageing process. Nature. 592, 695–703. 10.1038/s41586-021-03307-7 33911272 PMC9844150

[B177] Serrano M. Lin A. W. Mccurrach M. E. Beach D. Lowe S. W. (1997). Oncogenic ras provokes premature cell senescence associated with accumulation of p53 and p16INK4a. Cell. 88, 593–602. 10.1016/s0092-8674(00)81902-9 9054499

[B178] Sherr C. J. Mccormick F. (2002). The RB and p53 pathways in cancer. Cancer Cell. 2, 103–112. 10.1016/s1535-6108(02)00102-2 12204530

[B179] Shimizu I. Minamino T. (2019). Cellular senescence in cardiac diseases. J. Cardiol. 74, 313–319. 10.1016/j.jjcc.2019.05.002 31202488

[B180] Simpkin A. J. Hemani G. Suderman M. Gaunt T. R. Lyttleton O. Mcardle W. L. (2016). Prenatal and early life influences on epigenetic age in children: a study of mother-offspring pairs from two cohort studies. Hum. Mol. Genet. 25, 191–201. 10.1093/hmg/ddv456 26546615 PMC4690495

[B181] Sorrentino J. A. Krishnamurthy J. Tilley S. Alb J. G. JR. Burd C. E. Sharpless N. E. (2014). p16INK4a reporter mice reveal age-promoting effects of environmental toxicants. J. Clin. Invest. 124, 169–173. 10.1172/JCI70960 24334456 PMC3871242

[B182] Spach M. S. Starmer C. F. (1995). Altering the topology of gap junctions a major therapeutic target for atrial fibrillation. Cardiovasc Res. 30, 337–344. 10.1016/0008-6363(96)88514-2 7585823

[B183] Stein G. H. Drullinger L. F. Soulard A. Dulic V. (1999). Differential roles for cyclin-dependent kinase inhibitors p21 and p16 in the mechanisms of senescence and differentiation in human fibroblasts. Mol. Cell Biol. 19, 2109–2117. 10.1128/MCB.19.3.2109 10022898 PMC84004

[B184] Sun Z. Zhou D. Xie X. Wang S. Wang Z. Zhao W. (2016). Cross-talk between macrophages and atrial myocytes in atrial fibrillation. Basic Res. Cardiol. 111, 63. 10.1007/s00395-016-0584-z 27660282 PMC5033992

[B185] Sun L. Yan S. Wang X. Zhao S. Li H. Wang Y. (2017). Metoprolol prevents chronic obstructive sleep apnea-induced atrial fibrillation by inhibiting structural, sympathetic nervous and metabolic remodeling of the atria. Sci. Rep. 7, 14941. 10.1038/s41598-017-14960-2 29097705 PMC5668297

[B186] Sun X. Shi B. Zheng H. Min L. Yang J. Li X. (2018). Senescence-associated secretory factors induced by cisplatin in melanoma cells promote non-senescent melanoma cell growth through activation of the ERK1/2-RSK1 pathway. Cell Death Dis. 9, 260. 10.1038/s41419-018-0303-9 29449532 PMC5833767

[B187] Sykora M. Andelova K. Szeiffova Bacova B. Egan Benova T. Martiskova A. Knezl V. (2023). Hypertension induces pro-arrhythmic cardiac connexome disorders: protective effects of treatment. Biomolecules. 13, 330. 10.3390/biom13020330 36830700 PMC9953310

[B188] Takahashi A. Imai Y. Yamakoshi K. Kuninaka S. Ohtani N. Yoshimoto S. (2012). DNA damage signaling triggers degradation of histone methyltransferases through APC/C(Cdh1) in senescent cells. Mol. Cell. 45, 123–131. 10.1016/j.molcel.2011.10.018 22178396

[B189] Takahashi A. Loo T. M. Okada R. Kamachi F. Watanabe Y. Wakita M. (2018). Downregulation of cytoplasmic DNases is implicated in cytoplasmic DNA accumulation and SASP in senescent cells. Nat. Commun. 9, 1249. 10.1038/s41467-018-03555-8 29593264 PMC5871854

[B190] Testai L. Piragine E. Piano I. Flori L. Da Pozzo E. Miragliotta V. (2020). The citrus flavonoid naringenin protects the myocardium from ageing-dependent dysfunction: potential role of SIRT1. Oxid. Med. Cell Longev. 2020, 4650207. 10.1155/2020/4650207 32047577 PMC7003265

[B191] Thevaranjan N. Puchta A. Schulz C. Naidoo A. Szamosi J. C. Verschoor C. P. (2017). Age-associated microbial dysbiosis promotes intestinal permeability, systemic inflammation, and macrophage dysfunction. Cell Host Microbe. 21, 455–466 e4. 10.1016/j.chom.2017.03.002 28407483 PMC5392495

[B192] Timofeev O. Koch L. Niederau C. Tscherne A. Schneikert J. Klimovich M. (2020). Phosphorylation control of p53 DNA-binding cooperativity balances tumorigenesis and aging. Cancer Res. 80, 5231–5244. 10.1158/0008-5472.CAN-20-2002 32873634

[B193] Tong D. Schiattarella G. G. Jiang N. Daou D. Luo Y. Link M. S. (2022). Impaired AMP-activated protein kinase signaling in heart failure with preserved ejection fraction-associated atrial fibrillation. Circulation. 146, 73–76. 10.1161/CIRCULATIONAHA.121.058301 35858165 PMC9586456

[B194] Tran D. Bergholz J. Zhang H. He H. Wang Y. Zhang Y. (2014). Insulin-like growth factor-1 regulates the SIRT1-p53 pathway in cellular senescence. Aging Cell. 13, 669–678. 10.1111/acel.12219 25070626 PMC4118446

[B195] Travers J. G. Wennersten S. A. Pena B. Bagchi R. A. Smith H. E. Hirsch R. A. (2021). HDAC inhibition reverses preexisting diastolic dysfunction and blocks covert extracellular matrix remodeling. Circulation. 143, 1874–1890. 10.1161/CIRCULATIONAHA.120.046462 33682427 PMC8884170

[B196] Tsai C. F. Tai C. T. Hsieh M. H. Lin W. S. Yu W. C. Ueng K. C. (2000). Initiation of atrial fibrillation by ectopic beats originating from the superior vena cava: electrophysiological characteristics and results of radiofrequency ablation. Circulation. 102, 67–74. 10.1161/01.cir.102.1.67 10880417

[B197] Tsai Y. T. Lin F. Y. Lin C. S. Loh S. H. Li C. Y. Lin C. Y. (2019). B-type natriuretic peptide enhances fibrotic effects via matrix metalloproteinase-2 expression in the mouse atrium *in vivo* and in human atrial myofibroblasts *in vitro* . Transl. Res. 208, 30–46. 10.1016/j.trsl.2019.02.007 30857762

[B198] Unnikrishnan A. Freeman W. M. Jackson J. Wren J. D. Porter H. Richardson A. (2019). The role of DNA methylation in epigenetics of aging. Pharmacol. Ther. 195, 172–185. 10.1016/j.pharmthera.2018.11.001 30419258 PMC6397707

[B199] Van Der Velden H. M. Jongsma H. J. (2002). Cardiac gap junctions and connexins: their role in atrial fibrillation and potential as therapeutic targets. Cardiovasc Res. 54, 270–279. 10.1016/s0008-6363(01)00557-0 12062332

[B200] Van Deursen J. M. (2014). The role of senescent cells in ageing. Nature. 509, 439–446. 10.1038/nature13193 24848057 PMC4214092

[B201] Vanaja S. K. Russo A. J. Behl B. Banerjee I. Yankova M. Deshmukh S. D. (2016). Bacterial outer membrane vesicles mediate cytosolic localization of LPS and Caspase-11 activation. Cell. 165, 1106–1119. 10.1016/j.cell.2016.04.015 27156449 PMC4874922

[B202] Vellai T. Takacs-Vellai K. Zhang Y. Kovacs A. L. Orosz L. Muller F. (2003). Genetics: influence of TOR kinase on lifespan in *C. elegans* . Nature. 426, 620. 10.1038/426620a 14668850

[B203] Venetucci L. A. Trafford A. W. O'Neill S. C. Eisner D. A. (2008). The sarcoplasmic reticulum and arrhythmogenic calcium release. Cardiovasc Res. 77, 285–292. 10.1093/cvr/cvm009 18006483

[B204] Vezina C. Kudelski A. Sehgal S. N. (1975). Rapamycin (AY-22,989), a new antifungal antibiotic. I. Taxonomy of the producing streptomycete and isolation of the active principle. J. Antibiot. (Tokyo). 28, 721–726. 10.7164/antibiotics.28.721 1102508

[B205] Voigt N. Li N. Wang Q. Wang W. Trafford A. W. Abu-Taha I. (2012). Enhanced sarcoplasmic reticulum Ca2+ leak and increased Na+-Ca2+ exchanger function underlie delayed afterdepolarizations in patients with chronic atrial fibrillation. Circulation. 125, 2059–2070. 10.1161/CIRCULATIONAHA.111.067306 22456474 PMC4663993

[B206] Walden A. P. Dibb K. M. Trafford A. W. (2009). Differences in intracellular calcium homeostasis between atrial and ventricular myocytes. J. Mol. Cell Cardiol. 46, 463–473. 10.1016/j.yjmcc.2008.11.003 19059414

[B207] Wang R. Yu Z. Sunchu B. Shoaf J. Dang I. Zhao S. (2017). Rapamycin inhibits the secretory phenotype of senescent cells by a Nrf2-independent mechanism. Aging Cell. 16, 564–574. 10.1111/acel.12587 28371119 PMC5418203

[B208] Wang L. Y. Shen H. Yang Q. Min J. Wang Q. XI W. (2019). LncRNA-LINC00472 contributes to the pathogenesis of atrial fibrillation (Af) by reducing expression of JP2 and RyR2 via miR-24. Biomed. Pharmacother. 120, 109364. 10.1016/j.biopha.2019.109364 31562981

[B209] Wang L. Xu C. Johansen T. Berger S. L. Dou Z. (2021). SIRT1 - a new mammalian substrate of nuclear autophagy. Autophagy. 17, 593–595. 10.1080/15548627.2020.1860541 33292048 PMC8007159

[B210] Wang L. Wang B. Gasek N. S. Zhou Y. Cohn R. L. Martin D. E. (2022). Targeting p21(Cip1) highly expressing cells in adipose tissue alleviates insulin resistance in obesity. Cell Metab. 34, 186. 10.1016/j.cmet.2021.12.014 34986334 PMC8832725

[B211] Ward-Caviness C. K. (2021). Accelerated epigenetic aging and incident atrial fibrillation: new outlook on an immutable risk factor? Circulation. 144, 1912–1914. 10.1161/CIRCULATIONAHA.121.057533 34898242 PMC9070310

[B212] Watanabe S. Kawamoto S. Ohtani N. Hara E. (2017). Impact of senescence-associated secretory phenotype and its potential as a therapeutic target for senescence-associated diseases. Cancer Sci. 108, 563–569. 10.1111/cas.13184 28165648 PMC5406532

[B213] Wei Z. Bing Z. Shaohuan Q. Yanran W. Shuo S. Bi T. (2020). Expression of miRNAs in plasma exosomes derived from patients with atrial fibrillation. Clin. Cardiol. 43, 1450–1459. 10.1002/clc.23461 32940379 PMC7724226

[B214] Weichhart T. (2018). mTOR as regulator of lifespan, aging, and cellular senescence: a mini-review. Gerontology. 64, 127–134. 10.1159/000484629 29190625 PMC6089343

[B215] Wiersma M. Meijering R. A. M. Qi X. Y. Zhang D. Liu T. Hoogstra-Berends F. (2017). Endoplasmic reticulum stress is associated with autophagy and cardiomyocyte remodeling in experimental and human atrial fibrillation. J. Am. Heart Assoc. 6, e006458. 10.1161/JAHA.117.006458 29066441 PMC5721854

[B216] Wiersma M. Van Marion D. M. S. Wust R. C. I. Houtkooper R. H. Zhang D. Groot N. M. S. (2019). Mitochondrial dysfunction underlies cardiomyocyte remodeling in experimental and clinical atrial fibrillation. Cells. 8, 1202. 10.3390/cells8101202 31590355 PMC6829298

[B217] Wiley C. D. Velarde M. C. Lecot P. Liu S. Sarnoski E. A. Freund A. (2016). Mitochondrial dysfunction induces senescence with a distinct secretory phenotype. Cell Metab. 23, 303–314. 10.1016/j.cmet.2015.11.011 26686024 PMC4749409

[B218] Willems S. Klemm H. Rostock T. Brandstrup B. Ventura R. Steven D. (2006). Substrate modification combined with pulmonary vein isolation improves outcome of catheter ablation in patients with persistent atrial fibrillation: a prospective randomized comparison. Eur. Heart J. 27, 2871–2878. 10.1093/eurheartj/ehl093 16782716

[B219] Williams A. B. Schumacher B. (2016). p53 in the DNA-Damage-Repair process. Cold Spring Harb. Perspect. Med. 6, a026070. 10.1101/cshperspect.a026070 27048304 PMC4852800

[B220] Wolf I. Levanon-Cohen S. Bose S. Ligumsky H. Sredni B. Kanety H. (2008). Klotho: a tumor suppressor and a modulator of the IGF-1 and FGF pathways in human breast cancer. Oncogene. 27, 7094–7105. 10.1038/onc.2008.292 18762812

[B221] Wu J. J. Liu J. Chen E. B. Wang J. J. Cao L. Narayan N. (2013). Increased mammalian lifespan and a segmental and tissue-specific slowing of aging after genetic reduction of mTOR expression. Cell Rep. 4, 913–920. 10.1016/j.celrep.2013.07.030 23994476 PMC3784301

[B222] Wu W. Fu J. Gu Y. Wei Y. Ma P. Wu J. (2020). JAK2/STAT3 regulates estrogen-related senescence of bone marrow stem cells. J. Endocrinol. 245, 141–153. 10.1530/JOE-19-0518 32045363

[B223] Xiang L. Yangyang B. Ning Z. Changjian L. Yun X. Yue W. (2024). Senescent CD8+ T cells: a novel risk factor in atrial fibrillation. Cardiovasc. Res. 121, 97–112. 10.1093/cvr/cvae222 39382426

[B224] Xiao J. Sheng X. Zhang X. Guo M. Ji X. (2016). Curcumin protects against myocardial infarction-induced cardiac fibrosis via SIRT1 activation *in vivo* and *in vitro* . Drug Des. Devel Ther. 10, 1267–1277. 10.2147/DDDT.S104925 27099472 PMC4820283

[B225] Xie J. Chen Y. Hu C. Pan Q. Wang B. Li X. (2017). Premature senescence of cardiac fibroblasts and atrial fibrosis in patients with atrial fibrillation. Oncotarget. 8, 57981–57990. 10.18632/oncotarget.19853 28938531 PMC5601627

[B226] Xiong Y. Hannon G. J. Zhang H. Casso D. Kobayashi R. Beach D. (1993). p21 is a universal inhibitor of cyclin kinases. Nature. 366, 701–704. 10.1038/366701a0 8259214

[B227] Xu M. Tchkonia T. Kirkland J. L. (2016). Perspective: targeting the JAK/STAT pathway to fight age-related dysfunction. Pharmacol. Res. 111, 152–154. 10.1016/j.phrs.2016.05.015 27241018 PMC5026572

[B228] Yan J. Thomson J. K. Zhao W. Wu X. Gao X. Demarco D. (2018a). The stress kinase JNK regulates gap junction Cx43 gene expression and promotes atrial fibrillation in the aged heart. J. Mol. Cell Cardiol. 114, 105–115. 10.1016/j.yjmcc.2017.11.006 29146153 PMC5800987

[B229] Yan J. Zhao W. Thomson J. K. Gao X. Demarco D. M. Carrillo E. (2018b). Stress signaling JNK2 crosstalk with CaMKII underlies enhanced atrial arrhythmogenesis. Circ. Res. 122, 821–835. 10.1161/CIRCRESAHA.117.312536 29352041 PMC5924593

[B230] Yan J. Bare D. J. Desantiago J. Zhao W. Mei Y. Chen Z. (2021). JNK2, a newly-identified SERCA2 enhancer, augments an arrhythmic [Ca(2+)](SR) leak-load relationship. Circ. Res. 128, 455–470. 10.1161/CIRCRESAHA.120.318409 33334123 PMC7897290

[B231] Yi J. Luo J. (2010). SIRT1 and p53, effect on cancer, senescence and beyond. Biochim. Biophys. Acta. 1804, 1684–1689. 10.1016/j.bbapap.2010.05.002 20471503 PMC2989880

[B232] Yilmaz O. H. Katajisto P. Lamming D. W. Gultekin Y. Bauer-Rowe K. E. Sengupta S. (2012). mTORC1 in the paneth cell niche couples intestinal stem-cell function to calorie intake. Nature. 486, 490–495. 10.1038/nature11163 22722868 PMC3387287

[B233] Yoshikawa N. Tsuno N. H. Okaji Y. Kawai K. Shuno Y. Nagawa H. (2010). Isoprenoid geranylgeranylacetone inhibits human colon cancer cells through induction of apoptosis and cell cycle arrest. Anticancer Drugs. 21, 850–860. 10.1097/CAD.0b013e32833e53cf 20724917

[B234] Yoshimoto S. Loo T. M. Atarashi K. Kanda H. Sato S. Oyadomari S. (2013). Obesity-induced gut microbial metabolite promotes liver cancer through senescence secretome. Nature. 499, 97–101. 10.1038/nature12347 23803760

[B235] Yu E. Calvert P. A. Mercer J. R. Harrison J. Baker L. Figg N. L. (2013). Mitochondrial DNA damage can promote atherosclerosis independently of reactive oxygen species through effects on smooth muscle cells and monocytes and correlates with higher-risk plaques in humans. Circulation. 128, 702–712. 10.1161/CIRCULATIONAHA.113.002271 23841983

[B236] Zhang D. Wu C. T. Qi X. Meijering R. A. Hoogstra-Berends F. Tadevosyan A. (2014). Activation of histone deacetylase-6 induces contractile dysfunction through derailment of alpha-tubulin proteostasis in experimental and human atrial fibrillation. Circulation. 129, 346–358. 10.1161/CIRCULATIONAHA.113.005300 24146251

[B237] Zhang W. Song M. Qu J. Liu G. H. (2018). Epigenetic modifications in cardiovascular aging and diseases. Circ. Res. 123, 773–786. 10.1161/CIRCRESAHA.118.312497 30355081

[B238] Zhang D. Hu X. Li J. Liu J. Baks-Te Bulte L. Wiersma M. (2019). DNA damage-induced PARP1 activation confers cardiomyocyte dysfunction through NAD(+) depletion in experimental atrial fibrillation. Nat. Commun. 10, 1307. 10.1038/s41467-019-09014-2 30898999 PMC6428932

[B239] Zhang J. Ren D. Fedorova J. He Z. Li J. (2020). SIRT1/SIRT3 modulates redox homeostasis during ischemia/reperfusion in the aging heart. Antioxidants (Basel). 9, 858. 10.3390/antiox9090858 32933202 PMC7556005

[B240] Zhang J. Johnsen S. P. Guo Y. Lip G. Y. H. (2021). Epidemiology of atrial fibrillation: geographic/ecological risk factors, age, sex, genetics. Card. Electrophysiol. Clin. 13, 1–23. 10.1016/j.ccep.2020.10.010 33516388

[B241] Zhang Y. Zhang S. Li B. Luo Y. Gong Y. Jin X. (2022). Gut microbiota dysbiosis promotes age-related atrial fibrillation by lipopolysaccharide and glucose-induced activation of NLRP3-inflammasome. Cardiovasc Res. 118, 785–797. 10.1093/cvr/cvab114 33757127

[B242] Zhao H. Wu L. Yan G. Chen Y. Zhou M. Wu Y. (2021). Inflammation and tumor progression: signaling pathways and targeted intervention. Signal Transduct. Target Ther. 6, 263. 10.1038/s41392-021-00658-5 34248142 PMC8273155

[B243] Zheng D. L. Wu Q. R. Zeng P. Li S. M. Cai Y. J. Chen S. Z. (2022). Advanced glycation end products induce senescence of atrial myocytes and increase susceptibility of atrial fibrillation in diabetic mice. Aging Cell. 21, e13734. 10.1111/acel.13734 36278684 PMC9741501

[B244] Zhou X. Zhang H. He L. Wu X. Yin Y. (2018). Long-term l-Serine administration reduces food intake and improves oxidative stress and Sirt1/NFkappaB signaling in the hypothalamus of aging mice. Front. Endocrinol. (Lausanne). 9, 476. 10.3389/fendo.2018.00476 30190704 PMC6115525

[B245] Zhu H. Gao H. Ji Y. Zhou Q. Du Z. Tian L. (2022). Targeting p53-MDM2 interaction by small-molecule inhibitors: learning from MDM2 inhibitors in clinical trials. J. Hematol. Oncol. 15, 91. 10.1186/s13045-022-01314-3 35831864 PMC9277894

[B246] Zou D. Geng N. Chen Y. Ren L. Liu X. Wan J. (2016). Ranolazine improves oxidative stress and mitochondrial function in the atrium of acetylcholine-CaCl2 induced atrial fibrillation rats. Life Sci. 156, 7–14. 10.1016/j.lfs.2016.05.026 27208652

