# Supplementary material for: Atrial myocyte senescence as a driver of atrial fibrillation: mechanisms and therapeutic implications
Source: Front Cell Dev Biol. 2026 Jan 14;13:1702207. doi: 10.3389/fcell.2025.1702207 (PMC12846991; doi:10.3389/fcell.2025.1702207)
Supplement: Supplementary file 1 [file Table1.docx]

**Supplemental Table 1: Summary of research on the relationship between aging and atrial fibrillation.**

| **Research Model** | **Signaling Molecules** | **Signaling Pathways and Potential Relationship with AF** | **Ref.** |
| --- | --- | --- | --- |
| Rats, Humans | LPS, NLRP3 inflammasome | LPS + Glucose → NLRP3 inflammasome activation → Atrial fibrosis → AF↑ | (1) |
| HL-1 atrial myocytes | TNF-α, DNMT1, NF-κB, SERCA2a | TNF-α → DNMT1↑ → SERCA2a hypermethylation → SERCA2a↓ → AF↑ | (2) |
| Humans | NAD(P)H, ROS | NAD(P)H oxidase → ROS↑ → AF↑ | (3) |
| Human atrial myocytes, Drosophila | PARP-1, NAD+, ROS | Rapid pacing → Oxidative stress → DNA damage → PARP-1 overactivation → NAD+ depletion → Metabolic dysfunction → AF↑ | (4) |
| HL-1 atrial myocytes, Drosophila, AF patient tissues | MCU, Ca²⁺, ROS | Rapid electrical stimulation → Mitochondrial Ca²⁺ overload (via MCU) → Membrane potential collapse → ATP↓ → AF↑ | (5) |
| TNF-KO mice | TNF, IL-6, IL-1β, LPS | Aging → Gut dysbiosis → Increased permeability → LPS translocation → Systemic inflammation → AF↑ | (6) |
| HL-1 atrial myocytes | IL-1β, LPS, L-type Ca²⁺ channels | AF → Macrophage-derived IL-1β → L-type Ca²⁺ channel↓ → Effective refractory period↓ → AF↑ | (7) |
| In vitro reconstitution | LPS, LBP, CD14, TLR4-MD2 complex | LPS → LBP/CD14-dependent recognition → TLR4-MD2 activation → NF-κB/IRF3 → Pro-inflammatory cytokines → AF↑ | (8) |
| Mice, Human tumor tissues | TGF-β, IL-1α, NLRP3 | Senescent cardiomyocytes release TGF-β/IL-1α → Fibroblast activation → Atrial fibrosis → AF↑ | (9) |
| Mice | PI3K, TGF-β, IGF1R | PI3K inhibition → Cardiomyocyte apoptosis/fibrosis → AF↑ IGF1R activation → Physiological hypertrophy (no AF) | (10) |
| Aged human & rat atria, Sirt1-KO mice | Sirt1, RIPK1, MLKL complex | Sirt1↓ → RIPK1 acetylation↑ → RIPK1-RIPK3-MLKL necroptosis → Fibrosis → AF↑ | (11) |
| Mice | Nampt, NAD, RyR2, SERCA2a | Nampt↓ → NAD↓ → Sirtuins/SERCA2a↓ → RyR2 hyperphosphorylation → Ca²⁺ dysregulation → AF↑ | (12) |
| Human LAA, Mice | SIRT1, TGF-β/Smad | Homocysteine → SIRT1↓ → TGF-β/Smad↑ → Fibrosis → AF↑ | (13) |
| Human atria, Rabbits, HL-1 | PPAR-α, SIRT1, PGC-1α | PPAR-α/SIRT1/PGC-1α↓ → Mitochondrial dysfunction → Electrophysiological abnormalities → AF↑ | (14) |
| Mice | BNP, MMP-2, SIRT1 | BNP↑ → SIRT1 cytoplasmic redistribution → MMP-2↑ → Fibrosis → AF↑ | (15) |
| Rats | Ranolazine, NOX4, ROS, caspase-3 | Oxidative stress → NOX4 → Mitochondrial dysfunction → Caspase-3 activation → AF↑ | (16) |
| Human plasma | miRNAs, MAPK, mTOR | miR-92b-3p → MAPK↑ → AF↑ miR-1306-5p → mTOR↓ → ATP↓ → AF↑ | (17) |
| Human LAA, Rats | Pitx2 gene | Pitx2 promoter hypermethylation → Pitx2↓ → Fibrosis/oxidative stress → AF↑ | (18) |
| Human/mouse cardiomyocytes | LINC00472, RyR2, JP2 | LINC00472↓ → miR-24↑ → JP2↓ → RyR2 instability → Ca²⁺ leak → AF↑ | (19) |
| Human atria, Mice | miR-106b-25, RyR2 | miR-106b-25↓ → RyR2↑ → Ca²⁺ leak → Delayed afterdepolarizations (DADs) → AF↑ | (20) |
| Human atria, Mice | JNK2, CaMKII, RyR2 | Aging/stress → JNK2↑ → CaMKII↑ → RyR2 phosphorylation↑ → Ca²⁺ leak → DADs → AF↑ | (21) |
| Mice | RyR2, CaMKII | RyR2 mutation → Basal Ca²⁺ leak↑ → CaMKII↑ → AF↑ | (22) |
| Human right atria, HL-1 | NLRP3, CaMKII, RyR2 | Oxidative stress → NLRP3 priming → CaMKII↑ → RyR2 phosphorylation↑ → Ca²⁺ leak → AF↑ | (23) |
| Human right atrial myocytes | CaMKII, RyR2-S2814, NCX | Hypertension/aging/HF → CaMKII↑ → RyR2-S2814 phosphorylation↑ → NCX-mediated inward current → DADs → AF↑ | (24) |
| Mice | RyR2, CaMKII | CREM transgenic → RyR2-S2814 phosphorylation↑ → Ca²⁺ leak → DADs → AF↑ | (25) |
| Human atria, Mice | SPEG, RyR2, S2367 | SPEG↓ → RyR2-S2367 phosphorylation↓ → RyR2 hyperactivity → Ca²⁺ sparks↑ → DADs → AF↑ | (26) |
| HL-1 atrial myocytes | HDAC6, α-tubulin | Rapid pacing → HDAC6↑ → α-tubulin deacetylation → Cytoskeletal disruption → Ca²⁺ dysregulation → AF↑ | (27) |
| HL-1, Human LAA | Hsp27 | AF → Myolysis → Hsp27↑ → Cytoskeletal stabilization → AF↓ | (28) |
| HL-1 atrial myocytes, Canine isolated atrial myocytes | Hsp27 | Rapid pacing → Decreased calcium transient amplitude → Upregulation of HSP27 → Prevention of L-type calcium current reduction → AF↓ | (29) |
| HL-1 atrial myocytes | HSPB1, HDAC6, GGA*-59 | Rapid pacing → HDAC6↑ → GGA*-59 inhibits HDAC6 → Ca²⁺ transient recovery → AF↓ | (30) |
| HL-1 atrial myocytes | 4-PBA, HSPA5 | ER stress → Autophagy → Electrical/contractile dysfunction → AF↑ | (31) |
| Human atria, Rabbits, Mice | JNK, Cx43 | Aging/stress → JNK↑ → Cx43 transcription↓ → Impaired gap junctions → Conduction slowing → AF↑ | (32) |
| Mice | TGF-β1, RyR, SERCA2a | TGF-β1 → Fibrosis → Ca²⁺ dysregulation → Triggered activity → AF↑ | (33) |
| Mice | AMPK, mTOR, Cx43/Cx40 | AMPK↓ → mTOR↑ → Cx43/Cx40↓ → Electrical uncoupling → AF↑ | (34) |
| Human cardiac tissue | GJA5 gene, Cx40 | GJA5 mutation → Cx40 dysfunction → Conduction heterogeneity → Microreentry → AF↑ | (35) |
| Yorkshire swine | Cx40, Cx43 | Rapid pacing → Cx43↓ → Electrical uncoupling → AF↑ Cx40/Cx43 gene transfer → Conduction↑ → AF↓ | (36) |
| Human right atria, Mice | CaMKII, RyR2, NCX | CaMKII↑ → RyR2 phosphorylation↑ → NCX-mediated inward current → DADs → AF↑ | (37) |
| Human right atrial appendage | p53, p16, MMP-9 | Aging → p53/p16↑ → MMP-9↑ + eNOS↓ → Endothelial dysfunction → AF↑ | (38) |
| Human LAA | p16, SA-β-gal, TGF-β | AF → TGF-β/collagen↑ → p16/SA-β-gal↑ → Fibrosis → AF↑ | (39) |
| Mice, HL-1 | AGEs, p16, SA-β-gal | p16↑ → CDK4/6 inhibition → Rb activation → Cell cycle arrest → APD prolongation → early afterdepolarizations → AF↑ | (40) |
| Human LAA, HL-1 | p16, IL-1β/IL-6, RyR2, SERCA2a | AF → p16/p21/p53↑ → Senescence → IL-1β/IL-6↑ → Fibrosis → AF↑ | (41) |
| Mice | ACE | Aging → Na⁺ channel dysfunction → Conduction slowing → AF↓ ACE inhibitor → RAS inhibition → Conduction↑ → AF↓ | (42) |
| Human right atrial appendage | miR-199a-5p, p53 | p53 pathway activation → Cell cycle arrest/apoptosis → Cardiomyocyte dysfunction → AF↑ | (43) |

**Abbreviations:** 4-PBA, 4-phenylbutyric acid; ACE, angiotensin-converting enzyme; AF, atrial fibrillation; AGEs, advanced glycation end-products; AMPK, AMP-activated protein kinase; APD, action potential duration; ATP, adenosine triphosphate; BNP, B-type natriuretic peptide; Ca²⁺, calcium ion; CaMKII, calcium/calmodulin-dependent protein kinase II; CD14, cluster of differentiation 14; CDK4/6, cyclin-dependent kinase 4/6; CREM, cAMP-responsive element modulator; Cx40, connexin 40; Cx43, connexin 43; DADs, delayed afterdepolarizations; DNMT1, DNA methyltransferase 1; EADs, early afterdepolarizations; eNOS, endothelial nitric oxide synthase; ER, endoplasmic reticulum; GGA*-59, a synthetic HDAC6 inhibitor (exact chemical name unspecified); GJA5, gap junction protein alpha 5 (encodes Cx40); HF, heart failure; HDAC6, histone deacetylase 6; HSPA5, heat shock protein family A member 5; HSPB1, heat shock protein family B (small) member 1; Hsp27, heat shock protein 27; IGF1R, insulin-like growth factor 1 receptor; IL-1α/IL-1β, interleukin-1 alpha/beta; IL-6, interleukin-6; IRF3, interferon regulatory factor 3; JNK, c-Jun N-terminal kinase; JNK2, c-Jun N-terminal kinase 2; JP2, junctophilin-2; LAA, left atrial appendage; LBP, LPS-binding protein; LINC00472, long intergenic non-protein coding RNA 472; LPS, lipopolysaccharide; MAPK, mitogen-activated protein kinase; MCU, mitochondrial calcium uniporter; miR, microRNA; MMP-2/MMP-9, matrix metalloproteinase-2/-9; MLKL, mixed lineage kinase domain-like protein; mTOR, mechanistic target of rapamycin; NAD⁺, nicotinamide adenine dinucleotide (oxidized form); NAD(P)H, nicotinamide adenine dinucleotide (phosphate) (reduced form); NCX, sodium-calcium exchanger; NF-κB, nuclear factor kappa-light-chain-enhancer of activated B cells; NLRP3, NLR family pyrin domain containing 3 (inflammasome); NOX4, NADPH oxidase 4; PARP-1, poly(ADP-ribose) polymerase 1; PGC-1α, peroxisome proliferator-activated receptor gamma coactivator 1-alpha; PI3K, phosphatidylinositol 3-kinase; Pitx2, paired-like homeodomain 2; PPAR-α, peroxisome proliferator-activated receptor alpha; Rb, retinoblastoma protein; RIPK1/RIPK3, receptor-interacting serine/threonine-protein kinase 1/3; ROS, reactive oxygen species; RyR2, ryanodine receptor 2; SA-β-gal, senescence-associated beta-galactosidase; SERCA2a, sarcoplasmic/endoplasmic reticulum calcium ATPase 2a; SIRT1, sirtuin 1; Sirt1, sirtuin 1 (gene symbol); Smad, mothers against decapentaplegic homolog (TGF-β signaling); SPEG, striated muscle preferentially expressed protein kinase; TGF-β, transforming growth factor beta; TLR4-MD2, toll-like receptor 4-myeloid differentiation factor 2 complex; TNF, tumor necrosis factor; TNF-KO, tumor necrosis factor knockout; TNF-α, tumor necrosis factor alpha.

1. Zhang Y, Zhang S, Li B, Luo Y, Gong Y, Jin X, et al. Gut microbiota dysbiosis promotes age-related atrial fibrillation by lipopolysaccharide and glucose-induced activation of NLRP3-inflammasome. Cardiovasc Res. 2022;118(3):785-97.

2. Kao YH, Chen YC, Cheng CC, Lee TI, Chen YJ, Chen SA. Tumor necrosis factor-alpha decreases sarcoplasmic reticulum Ca2+-ATPase expressions via the promoter methylation in cardiomyocytes. Critical care medicine. 2010;38(1):217-22.

3. Kim YM, Guzik TJ, Zhang YH, Zhang MH, Kattach H, Ratnatunga C, et al. A myocardial Nox2 containing NAD(P)H oxidase contributes to oxidative stress in human atrial fibrillation. Circulation research. 2005;97(7):629-36.

4. Zhang D, Hu X, Li J, Liu J, Baks-Te Bulte L, Wiersma M, et al. DNA damage-induced PARP1 activation confers cardiomyocyte dysfunction through NAD(+) depletion in experimental atrial fibrillation. Nature communications. 2019;10(1):1307.

5. Wiersma M, van Marion DMS, Wust RCI, Houtkooper RH, Zhang D, Groot NMS, et al. Mitochondrial Dysfunction Underlies Cardiomyocyte Remodeling in Experimental and Clinical Atrial Fibrillation. Cells. 2019;8(10).

6. Thevaranjan N, Puchta A, Schulz C, Naidoo A, Szamosi JC, Verschoor CP, et al. Age-Associated Microbial Dysbiosis Promotes Intestinal Permeability, Systemic Inflammation, and Macrophage Dysfunction. Cell host & microbe. 2017;21(4):455-66 e4.

7. Sun Z, Zhou D, Xie X, Wang S, Wang Z, Zhao W, et al. Cross-talk between macrophages and atrial myocytes in atrial fibrillation. Basic research in cardiology. 2016;111(6):63.

8. Ryu JK, Kim SJ, Rah SH, Kang JI, Jung HE, Lee D, et al. Reconstruction of LPS Transfer Cascade Reveals Structural Determinants within LBP, CD14, and TLR4-MD2 for Efficient LPS Recognition and Transfer. Immunity. 2017;46(1):38-50.

9. Acosta JC, Banito A, Wuestefeld T, Georgilis A, Janich P, Morton JP, et al. A complex secretory program orchestrated by the inflammasome controls paracrine senescence. Nat Cell Biol. 2013;15(8):978-90.

10. Chen YC, Wijekoon S, Matsumoto A, Luo J, Kiriazis H, Masterman E, et al. Distinct functional and molecular profiles between physiological and pathological atrial enlargement offer potential new therapeutic opportunities for atrial fibrillation. 2024;138(15):22.

11. Jin X, Zhang Y, Zhou Y, Luo Y, Han X, Gao Y, et al. Sirt1 Deficiency Promotes Age-Related AF Through Enhancing Atrial Necroptosis by Activation of RIPK1 Acetylation. Circulation: Arrhythmia and Electrophysiology. 2024;17(7):e012452.

12. Feng D, Xu D, Murakoshi N, Tajiri K, Qin R, Yonebayashi S, et al. Nicotinamide Phosphoribosyltransferase (Nampt)/Nicotinamide Adenine Dinucleotide (NAD) Axis Suppresses Atrial Fibrillation by Modulating the Calcium Handling Pathway. International journal of molecular sciences. 2020;21(13).

13. Han L, Tang Y, Li S, Wu Y, Chen X, Wu Q, et al. Protective mechanism of SIRT1 on Hcy-induced atrial fibrosis mediated by TRPC3. Journal of cellular and molecular medicine. 2020;24(1):488-510.

14. Liu GZ, Hou TT, Yuan Y, Hang PZ, Zhao JJ, Sun L, et al. Fenofibrate inhibits atrial metabolic remodelling in atrial fibrillation through PPAR-alpha/sirtuin 1/PGC-1alpha pathway. British journal of pharmacology. 2016;173(6):1095-109.

15. Tsai YT, Lin FY, Lin CS, Loh SH, Li CY, Lin CY, et al. B-type natriuretic peptide enhances fibrotic effects via matrix metalloproteinase-2 expression in the mouse atrium in vivo and in human atrial myofibroblasts in vitro. Translational research : the journal of laboratory and clinical medicine. 2019;208:30-46.

16. Zou D, Geng N, Chen Y, Ren L, Liu X, Wan J, et al. Ranolazine improves oxidative stress and mitochondrial function in the atrium of acetylcholine-CaCl2 induced atrial fibrillation rats. Life sciences. 2016;156:7-14.

17. Wei Z, Bing Z, Shaohuan Q, Yanran W, Shuo S, Bi T, et al. Expression of miRNAs in plasma exosomes derived from patients with atrial fibrillation. Clinical cardiology. 2020;43(12):1450-9.

18. Donate Puertas R, Meugnier E, Romestaing C, Rey C, Morel E, Lachuer J, et al. Atrial fibrillation is associated with hypermethylation in human left atrium, and treatment with decitabine reduces atrial tachyarrhythmias in spontaneously hypertensive rats. Translational research : the journal of laboratory and clinical medicine. 2017;184:57-67 e5.

19. Wang LY, Shen H, Yang Q, Min J, Wang Q, Xi W, et al. LncRNA-LINC00472 contributes to the pathogenesis of atrial fibrillation (Af) by reducing expression of JP2 and RyR2 via miR-24. Biomedicine & pharmacotherapy = Biomedecine & pharmacotherapie. 2019;120:109364.

20. Chiang DY, Kongchan N, Beavers DL, Alsina KM, Voigt N, Neilson JR, et al. Loss of microRNA-106b-25 cluster promotes atrial fibrillation by enhancing ryanodine receptor type-2 expression and calcium release. Circ Arrhythm Electrophysiol. 2014;7(6):1214-22.

21. Yan J, Zhao W, Thomson JK, Gao X, DeMarco DM, Carrillo E, et al. Stress Signaling JNK2 Crosstalk With CaMKII Underlies Enhanced Atrial Arrhythmogenesis. Circulation research. 2018;122(6):821-35.

22. Chelu MG, Sarma S, Sood S, Wang S, van Oort RJ, Skapura DG, et al. Calmodulin kinase II-mediated sarcoplasmic reticulum Ca2+ leak promotes atrial fibrillation in mice. The Journal of clinical investigation. 2009;119(7):1940-51.

23. Heijman J, Muna AP, Veleva T, Molina CE, Sutanto H, Tekook M, et al. Atrial Myocyte NLRP3/CaMKII Nexus Forms a Substrate for Postoperative Atrial Fibrillation. Circulation research. 2020;127(8):1036-55.

24. Neef S, Dybkova N, Sossalla S, Ort KR, Fluschnik N, Neumann K, et al. CaMKII-dependent diastolic SR Ca2+ leak and elevated diastolic Ca2+ levels in right atrial myocardium of patients with atrial fibrillation. Circulation research. 2010;106(6):1134-44.

25. Li N, Chiang DY, Wang S, Wang Q, Sun L, Voigt N, et al. Ryanodine receptor-mediated calcium leak drives progressive development of an atrial fibrillation substrate in a transgenic mouse model. Circulation. 2014;129(12):1276-85.

26. Campbell HM, Quick AP, Abu-Taha I, Chiang DY, Kramm CF, Word TA, et al. Loss of SPEG Inhibitory Phosphorylation of Ryanodine Receptor Type-2 Promotes Atrial Fibrillation. Circulation. 2020;142(12):1159-72.

27. Zhang D, Wu CT, Qi X, Meijering RA, Hoogstra-Berends F, Tadevosyan A, et al. Activation of histone deacetylase-6 induces contractile dysfunction through derailment of alpha-tubulin proteostasis in experimental and human atrial fibrillation. Circulation. 2014;129(3):346-58.

28. Brundel BJ, Henning RH, Ke L, van Gelder IC, Crijns HJ, Kampinga HH. Heat shock protein upregulation protects against pacing-induced myolysis in HL-1 atrial myocytes and in human atrial fibrillation. Journal of molecular and cellular cardiology. 2006;41(3):555-62.

29. Brundel BJ, Shiroshita-Takeshita A, Qi X, Yeh YH, Chartier D, van Gelder IC, et al. Induction of heat shock response protects the heart against atrial fibrillation. Circulation research. 2006;99(12):1394-402.

30. Hu X, Li J, van Marion DMS, Zhang D, Brundel B. Heat shock protein inducer GGA*-59 reverses contractile and structural remodeling via restoration of the microtubule network in experimental Atrial Fibrillation. Journal of molecular and cellular cardiology. 2019;134:86-97.

31. Wiersma M, Meijering RAM, Qi XY, Zhang D, Liu T, Hoogstra-Berends F, et al. Endoplasmic Reticulum Stress Is Associated With Autophagy and Cardiomyocyte Remodeling in Experimental and Human Atrial Fibrillation. Journal of the American Heart Association. 2017;6(10).

32. Yan J, Thomson JK, Zhao W, Wu X, Gao X, DeMarco D, et al. The stress kinase JNK regulates gap junction Cx43 gene expression and promotes atrial fibrillation in the aged heart. Journal of molecular and cellular cardiology. 2018;114:105-15.

33. Choi EK, Chang PC, Lee YS, Lin SF, Zhu W, Maruyama M, et al. Triggered firing and atrial fibrillation in transgenic mice with selective atrial fibrosis induced by overexpression of TGF-beta1. Circulation journal : official journal of the Japanese Circulation Society. 2012;76(6):1354-62.

34. Tong D, Schiattarella GG, Jiang N, Daou D, Luo Y, Link MS, et al. Impaired AMP-Activated Protein Kinase Signaling in Heart Failure With Preserved Ejection Fraction-Associated Atrial Fibrillation. Circulation. 2022;146(1):73-6.

35. Gollob MH, Jones DL, Krahn AD, Danis L, Gong XQ, Shao Q, et al. Somatic mutations in the connexin 40 gene (GJA5) in atrial fibrillation. The New England journal of medicine. 2006;354(25):2677-88.

36. Igarashi T, Finet JE, Takeuchi A, Fujino Y, Strom M, Greener ID, et al. Connexin gene transfer preserves conduction velocity and prevents atrial fibrillation. Circulation. 2012;125(2):216-25.

37. Voigt N, Li N, Wang Q, Wang W, Trafford AW, Abu-Taha I, et al. Enhanced sarcoplasmic reticulum Ca2+ leak and increased Na+-Ca2+ exchanger function underlie delayed afterdepolarizations in patients with chronic atrial fibrillation. Circulation. 2012;125(17):2059-70.

38. Jesel L, Abbas M, Park SH, Matsushita K, Kindo M, Hasan H, et al. Atrial Fibrillation Progression Is Associated with Cell Senescence Burden as Determined by p53 and p16 Expression. Journal of clinical medicine. 2019;9(1).

39. Xie J, Chen Y, Hu C, Pan Q, Wang B, Li X, et al. Premature senescence of cardiac fibroblasts and atrial fibrosis in patients with atrial fibrillation. Oncotarget. 2017;8(35):57981-90.

40. Zheng DL, Wu QR, Zeng P, Li SM, Cai YJ, Chen SZ, et al. Advanced glycation end products induce senescence of atrial myocytes and increase susceptibility of atrial fibrillation in diabetic mice. Aging cell. 2022;21(12):e13734.

41. Adili A, Zhu X, Cao H, Tang X, Wang Y, Wang J, et al. Atrial Fibrillation Underlies Cardiomyocyte Senescence and Contributes to Deleterious Atrial Remodeling during Disease Progression. Aging and disease. 2022;13(1):298-312.

42. Jansen HJ, McRae MD, Belke DD, Rose RA. Chronic angiotensin-converting enzyme inhibition attenuates frailty and protects against atrial fibrillation in aging mice. Heart rhythm. 2024.

43. Chiang DY, Zhang M, Voigt N, Alsina KM, Jakob H, Martin JF, et al. Identification of microRNA-mRNA dysregulations in paroxysmal atrial fibrillation. International journal of cardiology. 2015;184:190-7.
